# Novel Antifungal Scaffold Targeting Tubulin Overcomes *Sclerotinia Sclerotiorum* Resistance

**DOI:** 10.1002/advs.202511492

**Published:** 2025-11-21

**Authors:** Lihui Shao, Xianqun Hu, Ying Wu, Xiang Zhou, Bo Zhang, Song Yang

**Affiliations:** ^1^ State Key Laboratory of Green Pesticide Key Laboratory of Green Pesticide and Agricultural Bioengineering Ministry of Education Center for R&D of Fine Chemicals of Guizhou University Guiyang Guizhou 550025 P. R. China; ^2^ School of Chemistry and Chemical Engineering Guizhou University Guiyang Guizhou 550025 P. R. China; ^3^ Shanghai Engineering Research Center of Green Energy Chemical Engineering College of Chemistry and Materials Science Shanghai Normal University 100 Guilin Road Shanghai 200234 P. R. China

**Keywords:** chromone‐acylhydrazone hybrids, fungal tubulin inhibition, pesticide resistance management, polyurethane microcapsules

## Abstract

The increasing prevalence of pesticide resistance in pathogenic bacteria, particularly among broad‐host‐range fungal pathogens such as *S. sclerotiorum*, poses a significant threat to global crop production and food security. Addressing this challenge requires the development of targeted compounds with novel mechanisms of action. Herein, a novel chromone‐acylhydrazone hybrid scaffold is designed and synthesized to specifically target fungal tubulin. Bioassay results identified compound G24 as a highly potent inhibitor of *S. sclerotiorum* (EC_50_ = 0.21 µg mL^−1^), exhibiting superior efficacy compared to conventional fungicides. Mechanistic investigations, including molecular docking, molecular dynamics, and immunofluorescence staining, revealed that G24 effectively disrupts tubulin polymerization by forming hydrogen bonds with key tubulin residues. Notably, G24 exhibits selective antifungal activity while maintaining mammalian safety, addressing critical toxicity concerns. To enhance field performance, polyurethane microcapsules loaded with G24 (G24‐Loaded PU‐MCs) are developed with an encapsulation efficiency of 89.41%, facilitating slow‐release kinetics, improved foliar adhesion, and prolonged pathogen suppression. This integrated approach, combining targeted compound design with microencapsulation, offers a promising and sustainable strategy for combating pesticide resistance and promoting global food security.

## Graphical abstract

Addressing pesticide resistance, novel chromone‐acylhydrazone hybrids targeting fungal tubulin are synthesized. Compound G24 potently inhibited *S. sclerotiorum* (EC_50_ = 0.21µg mL^−1^) and demonstrated enhanced field performance via microencapsulation, offering a sustainable strategy against resistance and for global food security.

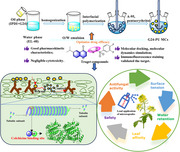

## 1 Introduction

Fungal diseases pose an escalating threat to global agricultural productivity.^[^ ^1^, ^2^, ^3^ ^]^ Among these, *Sclerotinia sclerotiorum* (*S. sclerotiorum*), a necrotrophic fungal pathogen with a broad host range, causes substantial economic losses in important crops such as soybean, oilseed rape (canola), sunflower, and a wide variety of vegetables. In severely affected fields, crop yields can be reduced by up to 80%,^[^ ^4^, ^5^, ^6^ ^]^ highlighting the urgent need for effective disease management strategies. *S. sclerotiorum* poses a significant threat to the rapeseed industry, causing substantial economic losses. This pathogen drastically reduces rapeseed yield and quality, impacting both seed production and oil content, leading to reduced farmer income. Crucially, infected rapeseed can be contaminated with mycotoxins like deoxynivalenol (DON) and fumonisins, posing serious health risks to humans and livestock. These toxins present a grave challenge to food safety, necessitating effective control strategies. Currently, chemical control remains the primary approach, with carbendazim (CB), a benzimidazole fungicide, being widely applied due to its targeting of fungal tubulin‐a critical protein for cellular processes, including cell division, intracellular transport, and maintenance of cell morphology. However, the overuse of CB has led to the emergence and proliferation of CB‐resistant *S. sclerotiorum* populations, necessitating the development of novel fungicides that effectively target tubulin and offer sustainable and resistance‐mitigating solutions.

The development of novel pharmaceuticals inspired by natural product scaffolds represents a significant paradigm in modern drug discovery.^[^ ^7^, ^8^, ^9^, ^10^ ^]^ Among these, the chromone skeleton, characterized by its fused *γ*‐pyrone and benzene rings, is a ubiquitous structural motif found in a diverse natural compounds. Tubulin, a crucial protein involved in microtubule dynamics, has emerged as a prominent therapeutic target for anti‐cancer, anti‐fungal, anti‐malarial, and anti‐viral applications.^[^ ^11^, ^12^, ^13^, ^14^, ^15^, ^16^ ^]^ While considerable researches have focused on chromone‐based tubulin‐targeting agents for anti‐tumor purposes, their potential in antifungal development remains underexplored. Concurrently, the hydrazone moiety has gained recognition as a versatile functional fragment in drug design due to its ability to coordinate and bind biological targets.^[^ ^17^, ^18^, ^19^, ^20^ ^]^ Notably, commercially available antifungal agents such as Ferimzone and Benquinox incorporate hydrazone functional groups, highlighting their potential in antifungal development. Additionally, hydrazone structures can form hydrogen bonds with amino acid residues in the tubulin binding pocket, enhancing biological activity and target affinity.^[^ ^21^, ^22^, ^23^, ^24^ ^]^ The exploration of novel fungicides combining chromone and hydrazone frameworks is thus of paramount importance, offering the potential for effective antifungal agents with novel mechanisms of action.

The efficacy of fungicide formulations is critical for disease control in agriculture. However, traditional fungicide application methods often result in environmental drawbacks, including pollution and ecosystem disruption. Due to drift, volatilization, and degradation, only ≈10% of applied fungicides reach the target pathogen, leading to low delivery efficiency.^[^ ^25^, ^26^, ^27^ ^]^ Consequently, there is a growing need for innovative technologies to address these limitations. Controlled‐release formulations, which encapsulate fungicides are within a carrier matrix, offers a promising solution. This approach enhances the stability and activity of active ingredient, reduces environmental pollution, and minimizes risks to non‐target organisms.^[^ ^28^ ^]^ Among the various materials investigated for controlled‐release coatings, polyurethane (PU) and polyurea (PU) stand out due to their biocompatibility, durability, tunable degradation rates, and favorable mechanical properties.^[^ ^29^ ^]^ Furthermore, the versatilities of PU materials allow for tailored release kinetics, providing a significant advantage over traditional formulations.

Guided by pesticide molecular design and microcapsule formulation principles, this study successfully synthesized twelve active hydrazone‐incorporated flavonoids (Figure S1, Supporting Information). Among these, compound G24 exhibited significant in vitro and in vivo efficacy against *S. sclerotiorum*. The mechanism of action of G24 was systematically investigated through microtubulin immunofluorescence assays, molecular docking, and molecular dynamics simulations. Additionally, the impact of G24 on cellular membrane integrity was assessed using scanning electron microscopy (SEM). To optimize the utilization efficiency of G24, we developed an innovative polyurethane microcapsule (PU MC) formulation encapsulating G24. The physicochemical properties of the MCs were thoroughly characterized using optical microscopy, SEM, Fourier transform infrared spectroscopy (FTIR), dynamic light scattering (DLS), contact angle measurements, and surface tension analysis. Finally, the interaction mechanisms between G24‐loaded PU MCs and rapeseed leaves were explored. This research offers a promising approach to the development of fungicides and their functional formulations, potentially improving pesticide utilization rates and effectively controlling microbial infections in agricultural settings.

## 2 Results and Discussion

### 2.1 Chemistry

A green and efficient synthetic strategy for the target compound G has been devised, employing a straightforward aldehyde‐amine condensation. Ethanol was selected as a benign and readily available solvent, while glacial acetic acid effectively catalyzed the reaction. The facile isolation of highly pure compound G was accomplished by recrystallization from the same ethanol solvent system. To elucidate the structure of compound G19, nuclear magnetic resonance (NMR) spectroscopy was performed. Spectroscopic analysis revealed key structural features of the synthesized compound. A prominent singlet at δ 11.39 ppm was observed, indicative of N─H proton within a benzoylhydrazine moiety (─NH─N═), suggesting the presence of this key structural element. The C‐2 proton of the γ‐pyranone moiety (─O─CH═) exhibited a resonance at δ 7.79 ppm. Additionally, a distinct resonance at δ 8.55 ppm, assigned to the proton adjacent to the imine bond (─CH = N─), corroborated the proposed connectivity. The seven aromatic protons of the substituted benzene ring appeared as a multiplet spanning δ 7.97–7.07 ppm, aligning with the deshielding effects of aromatic conjugation. The methoxycarbonyl methyl protons (CH_3_O‐) at the four‐position of benzoylhydrazine resonated as a singlet at δ 3.03 ppm. The peak at δ 3.00 ppm was assigned to the hydrogen on the 6‐isopropyl methyne carbon ((CH_3_)_2_CH–), while the two methyl groups within the isopropyl moiety are represented by double peaks at δ 1.19 ppm. The molecular formula was validated by high‐resolution mass spectrometry (HRMS), which yielded an [M + H]^+^ ion signal with a mass accuracy consistent with the calculated molecular weight.

### 2.2 In Vitro and In Vivo Antibacterial Activity of Target Compounds

**Figure 1:**
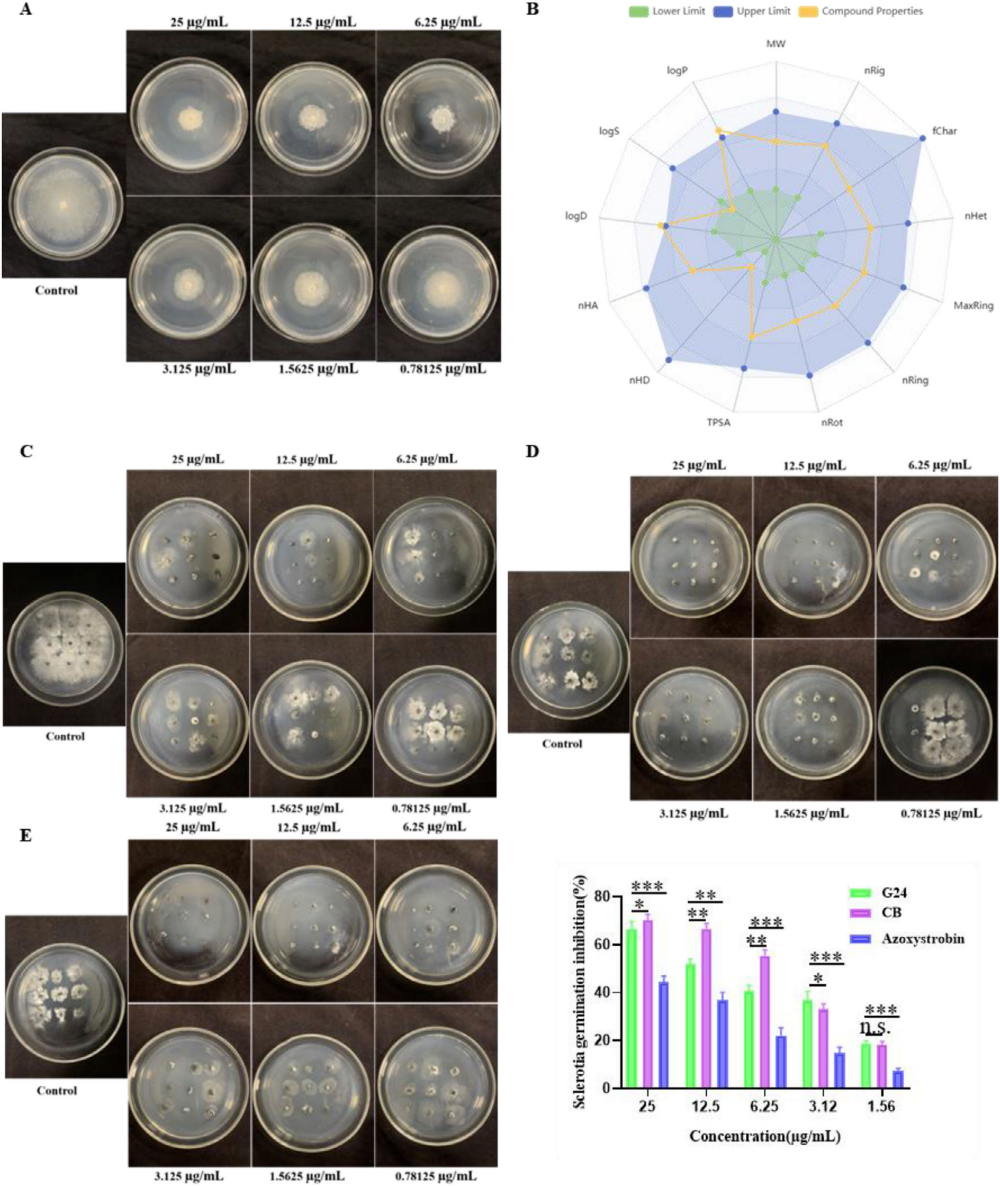
In vitro fungicidal activity of G24 against *S. sclerotiorum* A); Absorption, Distribution, Metabolism, Excretion, and Toxicity (ADMET) test B); inhibitory activities of compound G24 C), carbendazim D), and Azoxystrobin E) on the sclerotia germination of *S. sclerotiorum*. Statistically significant differences between the means were analyzed with one‐way ANOVA, followed by the least significant difference (LSD) post‐hoc test in (D) (For all studies, *n* = 3; ^*^ *p* < 0.05, ^**^ *p* < 0.01, ^***^ *p* < 0.001; n.s. = no significance).

Twelve compounds were evaluated for their bioactivity against *S. sclerotiorum* on canola. All compounds exhibited potent antifungal activity, with EC_50_ values ranging from 0.21 to 1.9 µg mL^−1^. Notably, compounds G18, G19, G21, G22, G23, G24, G25, and G26 demonstrated activity comparable to or exceeding that of the positive control fungicides CB, carbendazim suspension concentrate (CB‐SC), and Azoxystrobin. Structure‐activity relationship (SAR) analysis revealed that compounds with double substituents generally exhibited superior activity compared to those with single substituents (e.g., G23 > G26; G21, G24 > G15). Furthermore, fluorine substitution enhanced activity relative to chlorine substitution (e.g., G21 > G20, G24 > G23). In vitro assays also demonstrated strong antifungal activity of compound G24 against *S. sclerotiorum*, with an EC_50_ of 0.21 µg mL^−1^ (**Figure** **1**A, Table S1, Supporting Information). To assess the antimicrobial spectrum of G24, its inhibitory activity against strains of *Gibberella zeae*, *Alternaria alternata*, *Botryosphaeria dothidea*, *Fusarium oxysporum*, and *Verticillium dahlia* was further investigated. The results demonstrated that G24 exhibited significant inhibitory activity against all five fungal species at a concentration of 25 µg mL^−1^, comparable to the commercial fungicide carbendazim (Table S2, Supporting Information). Carbendazim‐resistant strain (*C‐S. sclerotiorum*) was used to test the antifungal effects of G24 on the resistant fungi. The results demonstratedthat G24 has appreciable inhibitory abilityto the resistant fungi, compared with carbendazim (Table S6, Supporting Information). On testing with *C‐S. sclerotiorum*, the inhibition by G24 was 4.5‐fold greater than that of carbendazim. These findings reveal that G24 engages distinct binding sites compared to carbendazim, positioning it as a promising lead compound for novel fungicide discovery. This divergence holds significant potential for developing new agents to combat carbendazim‐resistant fungal pathogens. As detailed in Table S3 (Supporting Information) and illustrated in Figure 9D,E, G24 provided a protective effect of 70.14% and a curative effect of 58.16% at a concentration of 100 µg mL^−1^. In addition, the efficacy of G24 was superior to or close to that of positive control, CB (curative rate: 62.96%; protective rate: 68.75%) and CB‐SC (curative rate: 47.18%; protective rate: 64.17%). Importantly, G24‐loaded PU‐MCs exhibited enhanced therapeutic efficacy (69.14%) and protective efficacy (88.19%) at the same concentration.

### 2.3 ADME Study

To comprehensively evaluate the drug‐likeness and potential off‐target toxicity of the lead compound, we assessed its absorption, distribution, metabolism, excretion, and toxicity (ADMET) profile using ADMET Lab 3.0 for pharmacokinetic analysis. The predicted ADMET properties are presented in Figure 1B and Table S4 (Supporting Information). The radar plot indicates that the physicochemical properties of G24 fall within acceptable ranges: molecular weight = 406.08, logS = ‐5.058, logP = 3.405, and logD = 3.196. The ADMET characteristics and drug properties of G24 can be summarized as follows:1) Absorption: Compound G24 exhibited negligible P‐glycoprotein (Pgp) inhibitory activity. Furthermore, it demonstrated favorable absorption characteristics, including high Caco‐2 permeability, substantial human intestinal absorption (HIA), and an estimated bioavailability ranging from 20% to 50%. 2) Distribution: G24 displayed a favorable volume of distribution and the potential to effectively cross the blood‐brain barrier (BBB). No inhibitory effects were observed on the liver‐specific transporters OATP1B1 and OATP1B3. 3) Metabolism: G24 did not inhibit CYP2D6, CYP3A4, or CYP2B6. However, it exhibited inhibitory activity against CYP2C19. 4) Excretion: G24 demonstrated a high clearance rate and a short half‐life. 5) Toxicological Safety: G24 presented a favorable toxicological profile, indicating a minimal risk of eye corrosion and skin irritation. Low oral acute toxicity, ototoxicity, A549 cytotoxicity, and drug‐induced neurotoxicity were predicted in rats. 6) Drug‐likeness: Notably, G24 adheres to Lipinski's Rule of Five, Pfizer's Rule, and the Golden Triangle principle, suggesting advantageous ADMET properties. In summary, G24 exhibits promising pharmacokinetic properties, warranting further investigation and development in future studies.

### 2.4 Sclerotia Germination Inhibition by Compound G24

*S. sclerotiorum* relies on sclerotia, dormant structures arising from the aggregation of vegetative mycelia, for survival in harsh environments and as a primary means of initiating plant disease. The remarkable longevity of sclerotia, coupled with their role as initial inoculum, presents a major obstacle to effective disease control. Therefore, targeting sclerotial development, specifically inhibiting germination, is a critical approach for managing *S. sclerotiorum* infections. The impact of compound G24 on sclerotial germination is illustrated in Figure 1C–E. At concentrations of 25, 12.5, 6.25, 3.12, and 1.56 mg mL^−1^, compound G24 inhibited sclerotial germination by 66.67%, 51.85%, 40.74%, 37.04%, and 18.92%, respectively. For comparison, the inhibition rates achieved with CB at the same concentrations were 70.37%, 66.67%, 55.56%, 33.33%, and 18.52%. These data indicate that compound G24 ability to suppress sclerotial germination is similar to or greater than that of CB, and more effective than azoxystrobin at the tested concentrations. Collectively, these experimental results demonstrate that compound G24 exhibits a degree of inhibitory activity against sclerotial germination. This suggests that compound G24 could potentially limit the production and dispersal of primary inoculum, thereby contributing to the control of this pathogen.

### 2.5 Morphological Studies

**Figure 2:**
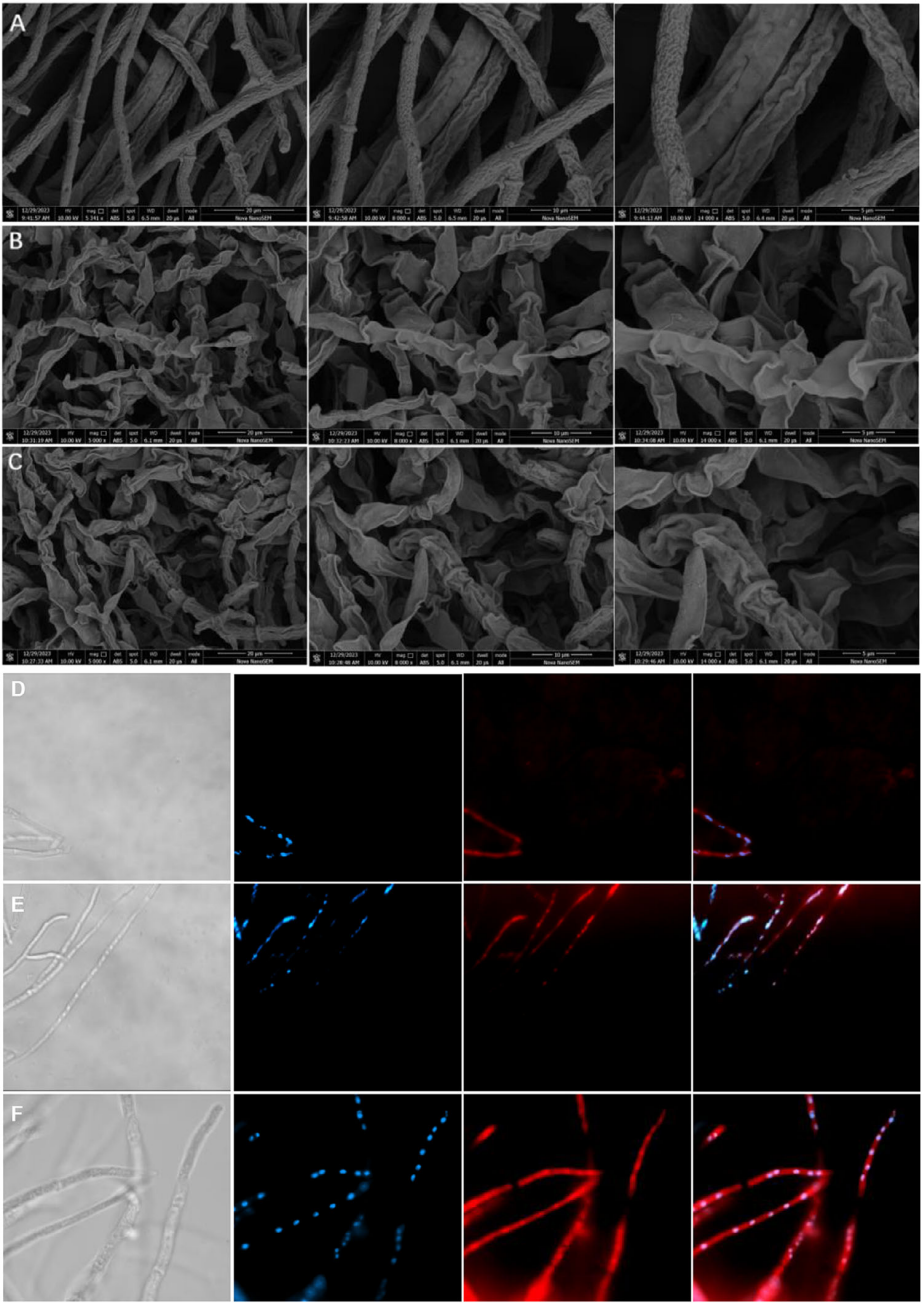
The effects of G24 at different concentrations on the morphology A: 0 µg mL^−1^; B: 25 µg mL^−1^; C: 12.5 µg mL^−1^); Effects of G24 on the cellular microtubule network were visualized by immunofluorescence assay D: 3.125 µg mL^−1^, E: 6.25 µg mL^−1^, F: 0 µg mL^−1^).

SEM analysis demonstrated that G24 significantly impacts the cell membrane integrity of *S. sclerotiorum*. Untreated cells exhibited a smooth, unperturbed surface. However, exposure to increasing concentrations of G24 resulted in a marked shift in cell morphology, including the development of a corrugated texture, cellular shrinkage, and eventual collapse. These G24‐induced alterations, visualized in **Figure** **2**A–C, indicate a potent interaction between the compound and the pathogen, leading to cellular damage.

### 2.6 Microtubule Antibody Staining

The tubulin‐microtubule system plays a pivotal role in maintaining cellular architecture and orchestrating fundamental cellular processes. To elucidate the effects of compound G24 on microtubule dynamics in fungal cells, we employed immunofluorescence microscopy analysis. In control specimens, as illustrated in Figure 2D–F, fungal cells demonstrated a characteristic microtubule network organization, featuring well‐organized, filamentous microtubules that exhibited uniform perinuclear distribution. However, following an 8 h exposure to G24, substantial structural alterations were observed in the perinuclear tubulin architecture, indicative of significant microtubule disruption. Particularly noteworthy was the observation that at a G24 concentration of 6.25 µg mL^−1^, a marked microtubule retraction was evident in the perinuclear region, concomitant with the emergence of numerous disorganized, punctate structures that were distinctly observable.

### 2.7 Molecular Modeling Study

**Figure 3:**
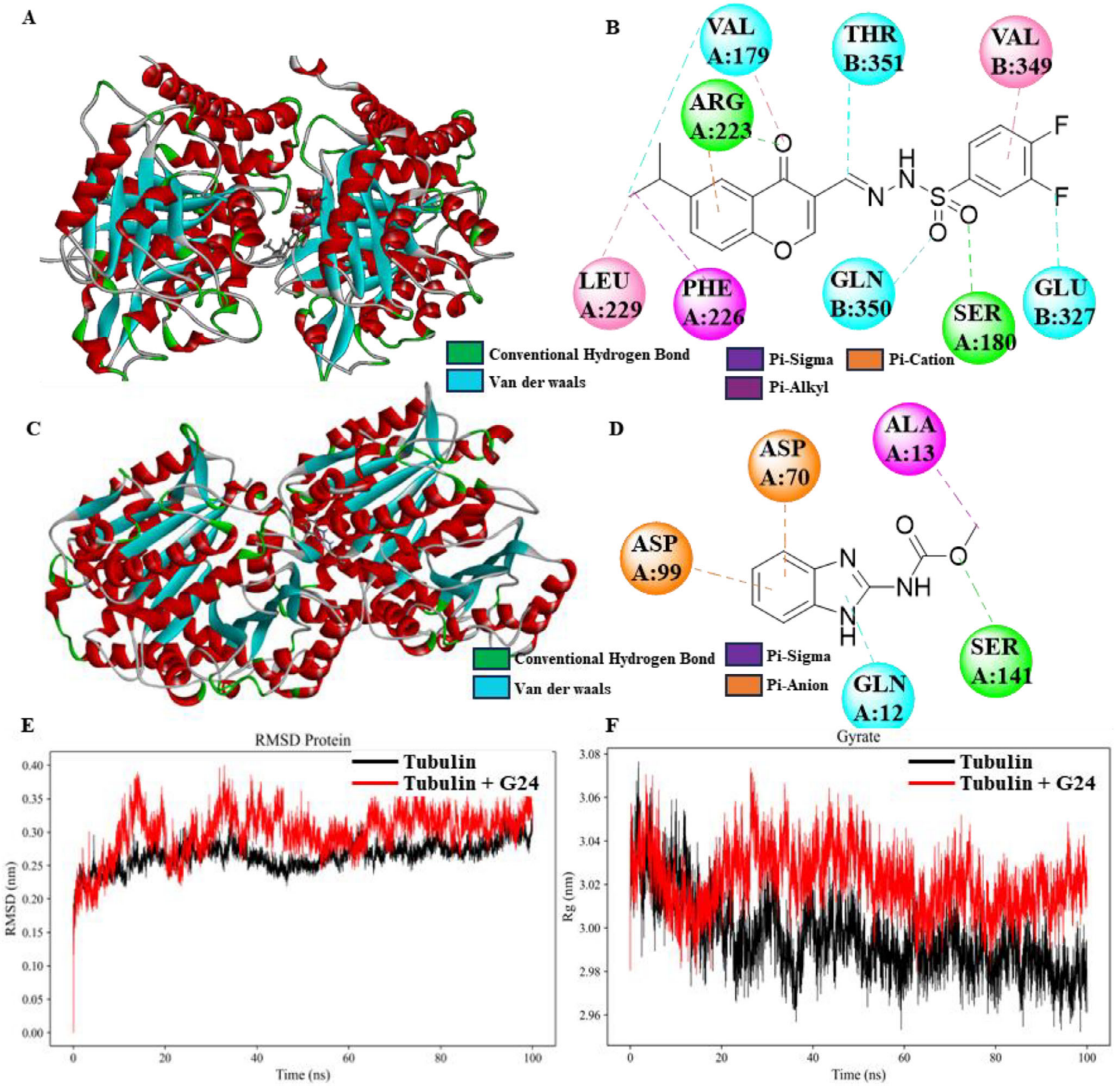
Docking modes of compound G24 A,B) and carbendazim C,D); Molecular dynamics simulation of G24 with tubulin E) Root‐Mean‐Square Deviation (RMSD); F) Radius of Gyration (Rg).

G24 exhibited the most potent anti‐proliferative activity in the initial screening and effectively inhibited tubulin polymerization, leading to its selection as a lead compound for further investigation. To elucidate the molecular basis of the interaction between G24 and the microtubule system, we performed molecular modeling studies. Docking analysis revealed that the 3‐fluorobenzoyl hydrazine moiety of G24 occupied the aqualoid‐binding pocket of *β*‐tubulin (**Figure** **3**A,B). This analysis identified three key interactions: 1) Hydrophobic residues ARG223 and Ser180 stabilized the complex via two hydrogen bonds, which appear critical for binding affinity. 2) The 3,4‐difluorobenzyl sulfonyl hydrazone and chromone core nucleocarbonyl groups formed C─H bonds with residues GLN350, THR351, GLU327, and VAL179, respectively, underscoring the structural importance of this motif in modulating biological activity. 3) The isopropyl group on the chromone core established Pi‐Alkyl interactions with residues LEU229 and VAL179, and a Pi‐Sigma interaction with residue PHE226. Importantly, the binding conformation of G24 demonstrated spatial complementarity with classical tubulin inhibitors, such as colchicine, within the hydrophobic cavity of the *β*‐tubulin subunit.

In a comparative analysis, molecular docking studies were performed to assess the binding affinity of CB to *S. sclerotiorum* tubulin (Figure 3C,D). The results revealed that the ‐OCH_3_ group of the benzimidazole moiety forms hydrogen bonds with Ser141 of the tubulin protein. These findings, in conjunction with previous data, suggest a stronger interaction between compound G24 and tubulin compared to CB. Collectively, the molecular docking results provide compelling evidence that the designed novel compounds exhibit favorable binding interactions with tubulin, supporting their potential as tubulin inhibitors.

### 2.8 Molecular Dynamics Analysis

Root‐Mean‐Square Deviation (RMSD): Molecular dynamics simulations of alpha‐beta‐tubulin systems, with and without ligand binding, exhibited RMSD values ranging from 0.10 to 0.40 nm, indicating overall structural stability. The ligand‐free protein (black curve) maintained a relatively stable RMSD ≈0.25 nm. Conversely, the G24‐bound system (red curve) displayed slightly elevated RMSD values (Figure 3E). Specifically, the ligand‐free protein RMSD rapidly increased from 0 to ≈0.20 nm within the initial 10 ns, followed by fluctuations within a narrow range of 0.23–0.27 nm, demonstrating consistent dynamic behavior. In contrast, the G24‐bound system exhibited the largest RMSD fluctuations (0.28–0.38 nm) after initial stabilization, suggesting a more pronounced impact of G24 on protein dynamics. In summary, the ligand‐free protein system demonstrated the highest stability in the absence of ligands, while the G24‐bound system exhibited the highest RMSD values and the greatest degree of fluctuation, potentially inducing significant conformational adaptations within the protein structure.

Radius of Gyration (Rg) Analysis: A smaller radius of gyration (Rg) is indicative of a more compact and stable system, suggesting stronger intramolecular interactions. Figure 3F illustrates the temporal evolution of the Rg for the ligand‐free alpha‐beta‐tubulin and its complexes with ligand G24. The mean Rg for the ligand‐free protein (black curve) was ≈3.00 nm, with fluctuations ranging from 2.96 to 3.06 nm, indicating inherent structural flexibility and dynamic behavior in the absence of ligands. The mean Rg for the G24 complex (red curve) was slightly higher than that of the ligand‐free protein, at ≈3.02 nm, with fluctuations ranging from 2.97 to 3.08 nm. These larger fluctuations suggest that G24 binding may introduce additional flexibility, resulting in a slightly more expanded overall protein structure. In summary, G24 binding appears to modestly increase protein flexibility, potentially leading to a less compact conformation.

**Figure 4:**
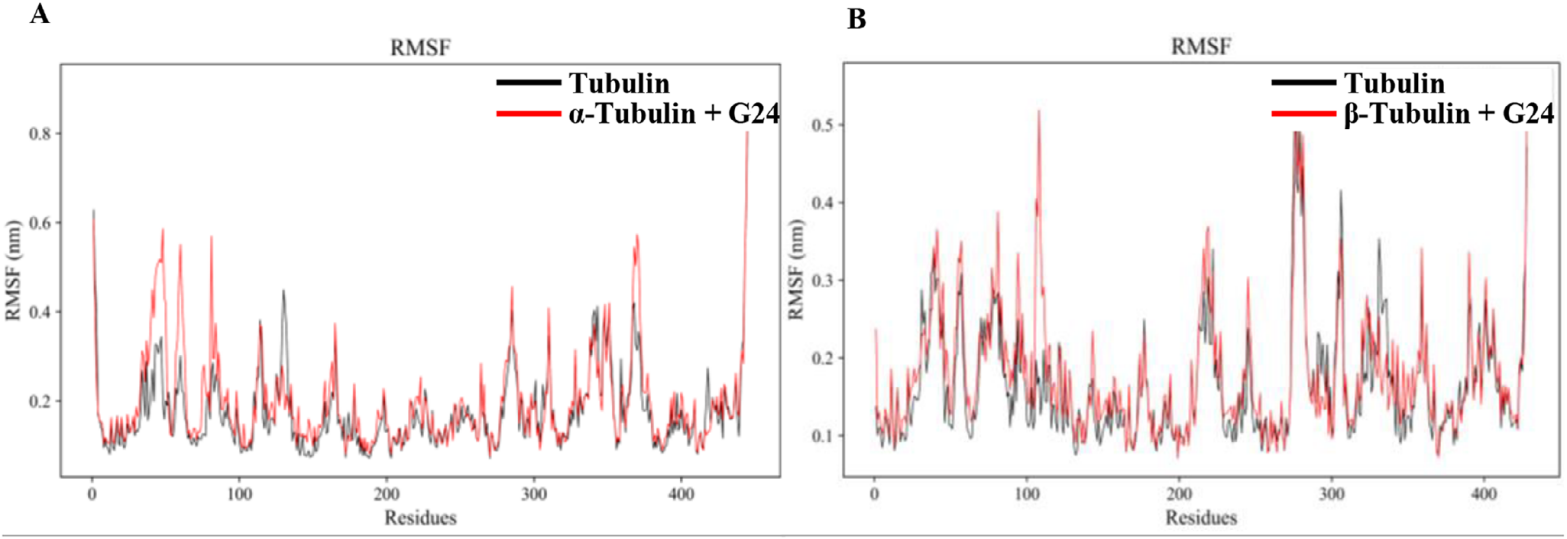
A,B) Root‐Mean‐Square Fluctuation (RMSF).

Root‐Mean‐Square Fluctuation (RMSF): Analysis of residue‐specific fluctuations, as depicted in **Figure** **4**A,B, provides insights into the dynamic behavior of alpha and beta‐tubulin and the influence of ligand binding on their stability. In alpha‐tubulin (Figure 4A), the ligand‐free protein (black) exhibited fluctuations ranging from 0.1 to 0.6 nm, with particularly pronounced fluctuations in the regions corresponding to residues 0–50 and 400–450. The latter region displayed fluctuations approaching 0.8 nm, indicative of high flexibility. The G24‐bound system (red) showed the most substantial fluctuations, particularly in the regions encompassing residues 0–100 and 300–400, with the latter fluctuating between 0.6 and 0.65 nm. In beta‐tubulin (Figure 4B), the ligand‐free protein (black) fluctuated between 0.1 and 0.7 nm, with notable fluctuations near residue 300 and in the 400–450 residue region, where fluctuations approached 1.0 nm. The G24‐bound system (red) exhibited elevated fluctuations, particularly in the 100–120 residue region, approaching 0.55 nm. Collectively, these data indicate that specific residue regions within both alpha‐ and beta‐tubulin exhibit inherent flexibility in the ligand‐free protein state, notably residues 0–50 and 400–450 in alpha‐tubulin and residues 300 and 400–450 in beta‐tubulin. Ligand binding, specifically G24, exerted the most significant influence on the fluctuations of residues 0–100 and 300–400 in alpha‐tubulin and residues 100–120 in beta‐tubulin, potentially increasing protein flexibility in these regions.

**Figure 5:**
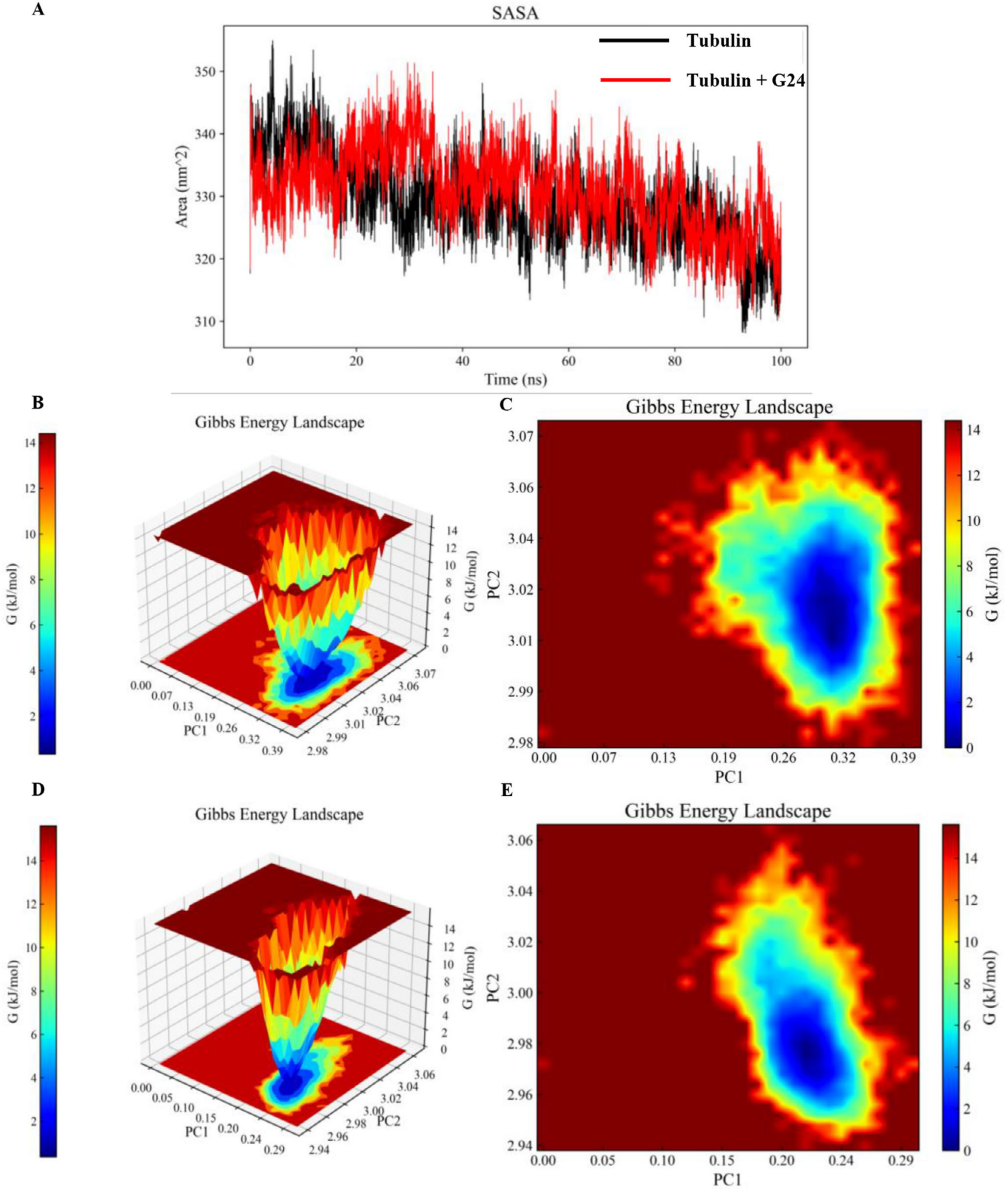
Molecular dynamics simulation of G24 with tubulin: A) Solvent Accessible Surface Area (SASA); B,C) 3D Gibbs free energy landscape of G24‐tubulin complex; D,E) 3D Gibbs free energy landscape of tubulin.

Solvent Accessible Surface Area (SASA) Analysis: Solvent accessible surface area (SASA) quantifies the surface area of a protein exposed to the solvent, providing insights into conformational changes and interactions with small molecules (**Figure** **5**A). Analysis of the temporal evolution of SASA revealed a generally consistent trend between the ligand‐free protein alpha‐beta‐tubulin (black curve) and the G24‐bound complex (red curve), with no significant differences observed. The mean SASA values for both the ligand‐free protein and the ligand‐bound system were similar, ≈330 nm^2^. Notably, during the later stages of the simulation (60–100 ns), the SASA curves for both systems converged, further suggesting that ligand binding did not substantially alter the overall solvent accessibility of the protein. These results indicate that while ligand binding may induce localized structural changes, its impact on the global solvent‐accessible surface area of the protein is limited.

Gibbs Free Energy Landscape Analysis: To further elucidate the stability of the complex, we constructed a 3D free energy landscape using the first two principal components (PC1 and PC2) derived from principal component analysis (PCA) of RMSD and Rg values for tubulin and G24, with the relative Gibbs free energy as the *Z*‐axis. The free energy landscape reveals that the minimum free energy for the ligand‐free protein is concentrated near PC1 = 0.20 and PC2 = 2.98, with a relatively narrow distribution (Figure 5B,C). The minimum free energy distribution for the G24‐bound system was slightly broader (Figure 5D,E), concentrated within the range of PC1 = 0.25‐0.37 and PC2 = 2.96‐3.04. The expansion of the free energy distribution upon ligand binding, particularly with G24, suggests that ligand binding increases the conformational diversity of the system, potentially influencing the dynamic behavior and stability of the protein.

### 2.9 Cytotoxicity

The cytotoxicity of compound G24 was evaluated using MTT assays on NRK and SV‐HUC‐1 cell lines, with 5‐Fluorouracil (5‐FU) included as a positive control. The results, summarized in Table S5 (Supporting Information), indicate that G24 consistently induced lower cytotoxicity than 5‐FU across a range of concentrations (6.25, 12.5, 25, 50, and 100 µmol L^−1^). Interestingly, at a concentration of 100 µmol L^−1^, the inhibitory effect of G24 on NRK cells was similar to that observed with 5‐FU. Importantly, despite demonstrating robust antifungal activity both in vivo and in vitro, G24 exhibited only limited cytotoxicity, suggesting a promising safety profile for potential therapeutic applications.

To rigorously validate the observation that G24 exhibits reduced toxicity against mammals and *Sclerotinia sclerotiorum* due to selective targeting of tubulin, we present a comprehensive explanation grounded in molecular docking model results. Our findings elucidate the differential binding affinities and interaction patterns of G24 with tubulin homologs across species, providing a mechanistic basis for its observed selectivity. To mammalian tubulin (PDB: 1SA0), G24 forms a crucial hydrogen bond with Gln11. The chromone core's phenyl ring engages in ππ‐alkyl interactions with Ala316, Leu248, and Lys352, complemented by an electrostatic interaction with Lys254 (Figure 2S). A comparative analysis of G24's docking poses with mammalian tubulin against those with *Sclerotinia sclerotiorum* tubulin (Figure 3A,B) reveals significant differences in binding characteristics. 1) Reduced Hydrogen Bonding with Mammalian Tubulin: G24 forms a lower number of hydrogen bonds with mammalian tubulin compared to its interactions with *Sclerotinia sclerotiorum* tubulin; 2) Limited Interaction Diversity with Mammalian Tubulin: The variety of interaction types between G24 and mammalian tubulin (Conventional Hydrogen Bond, Pi‐Alkyl, and Attractive Charge) is less diverse than that observed with Sclerotinia sclerotiorum tubulin (Conventional Hydrogen Bond, Pi‐Alkyl, Pi‐Sigma, Pi‐Cation, and Van der waals). Collectively, these molecular docking simulations demonstrate a discernible selectivity of G24 toward different tubulin isoforms. The observed lower binding affinity of G24 to mammalian tubulin directly correlates with and provides a mechanistic explanation for our experimental findings of reduced mammalian cell cytotoxicity. This differential binding propensity highlights G24's potential as a targeted therapeutic agent.

### 2.10 Characterization of G24‐Loaded PU‐MCs

**Figure 6:**
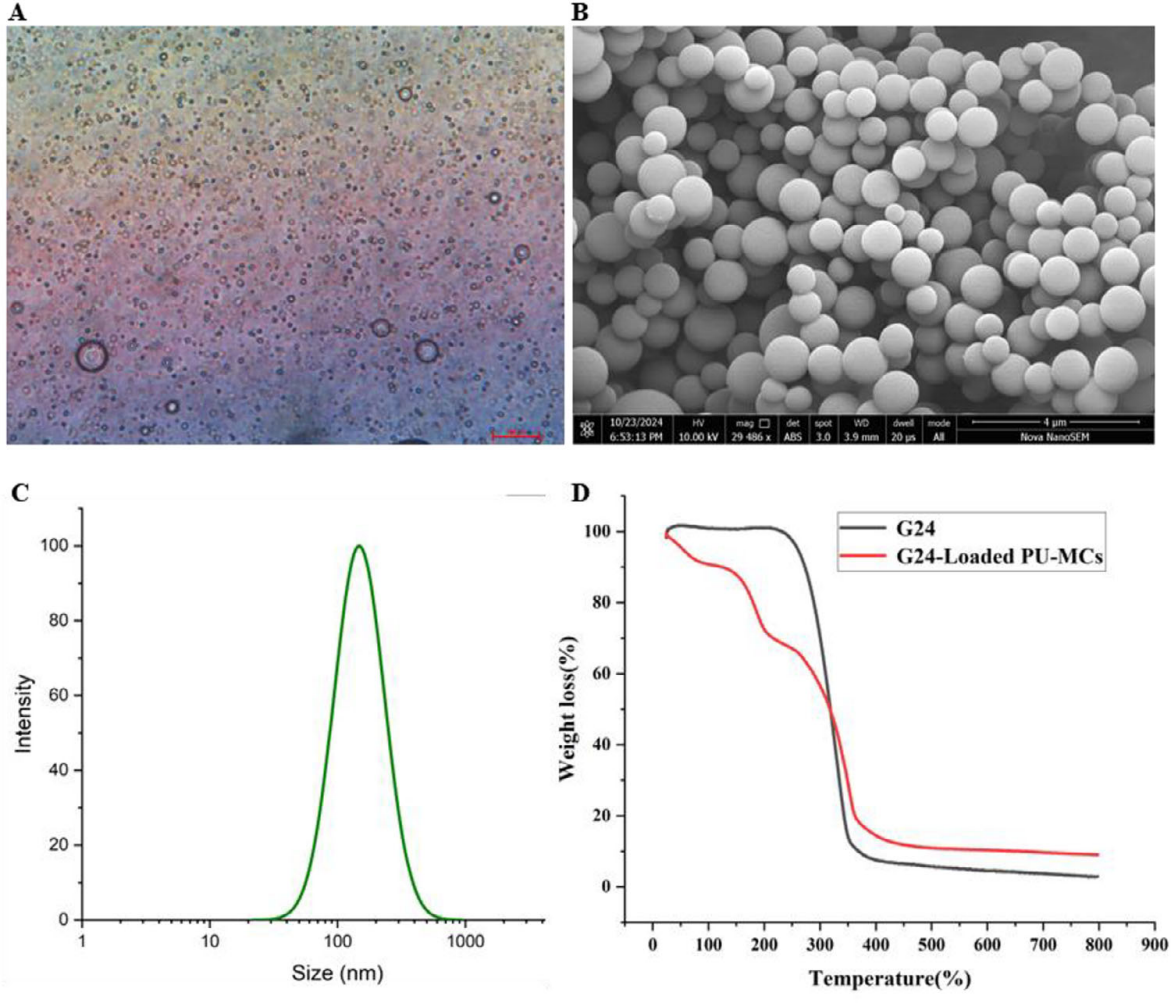
A) Optical micrograph; B) Scanning electron microscopy (SEM) images; C) Particle size distribution of G24‐loaded PU‐MCs; D) Thermogravimetric analysis (TGA) curves of G24 and G24‐loaded PU‐MCs.

**Figure 7:**
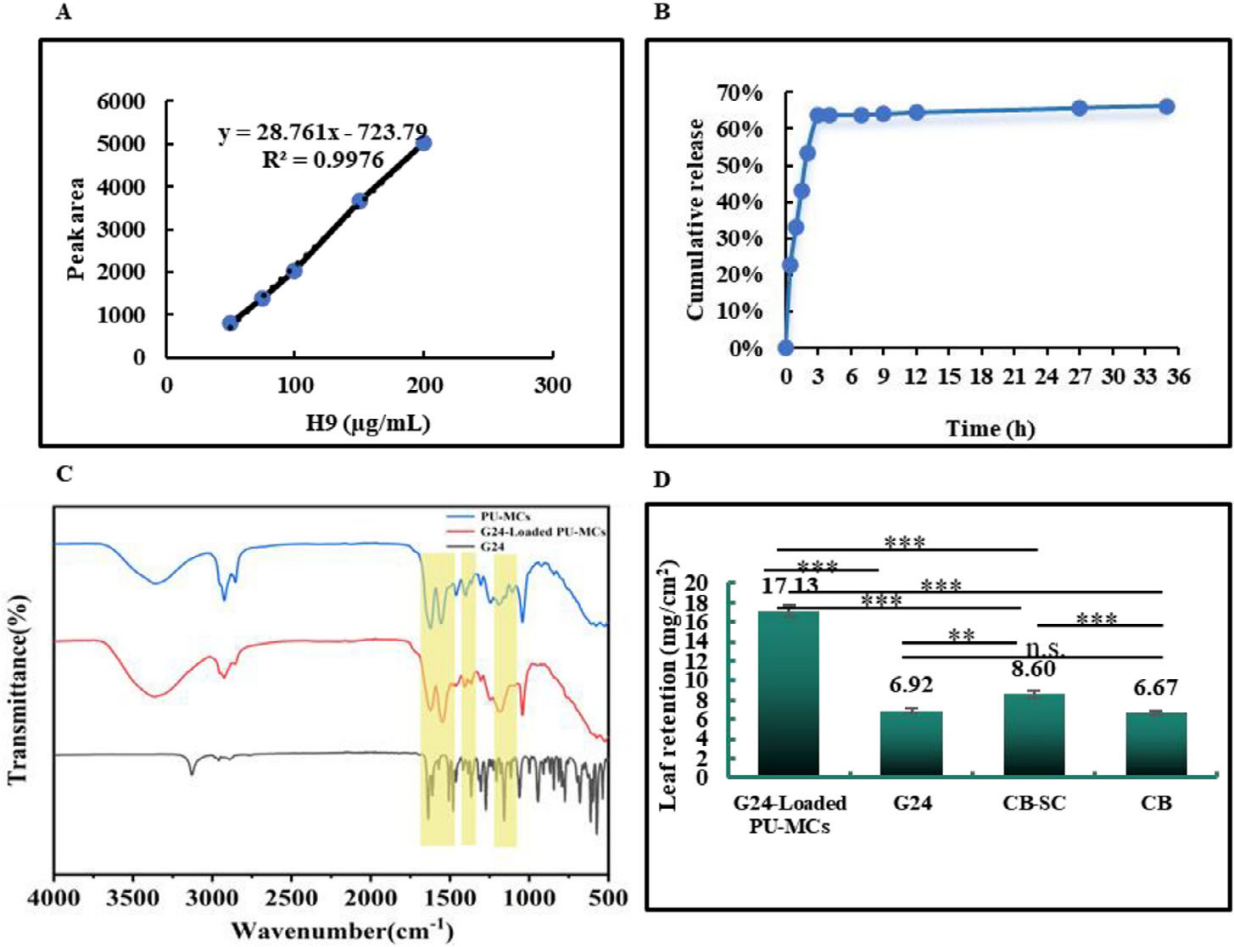
A) High‐performance liquid chromatography (HPLC) standard curve for G24 (peak time: 4.2 min); B) G24 release rate‐time curves of the prepared G24‐loaded PU‐MCs; C) Fourier‐transform infrared (FT‐IR) spectrum of G24, empty PU‐MCs, and G24‐loaded PU‐MCs; D) Leaf retention of G24‐loaded PU‐MCs suspension on rape leaf. Statistically significant differences between the means were analyzed with one‐way ANOVA, followed by the least significant difference (LSD) post‐hoc test in (D) (For all studies, *n* = 3; ^*^ *p* < 0.05, ^**^ *p* < 0.01, ^***^ *p* < 0.001; n.s. = no significance).

The morphology of G24‐loaded PU‐MCs was characterized using scanning electron microscopy (SEM) and optical microscopy (**Figure** **6**A,B). The micrographs revealed that the microcapsules possessed a consistently spherical morphology, exhibiting high monodispersity and minimal aggregation in aqueous suspensions. Fourier‐transform infrared (FT‐IR) spectroscopy (**Figure** **7**C) identified characteristic absorption bands at 1637 and 1548 cm^−1^, attributable to the stretching vibrations of ─C═O and ─CONH‐ groups, respectively. Additional peaks at 1401 and 1190 cm^−1^ were assigned to ─C─N─ and ‐C─O─C─ bonds. The absence of any novel absorption bands indicated the physical encapsulation of G24 within the PU‐MCs, suggesting the lack of chemical interactions between the drug and the polymer matrix. Dynamic light scattering (DLS) analysis (Figure 6C) revealed a hydrodynamic diameter distribution ranging from 58 to 981 nm, with a primary distribution centered at 147.6 nm, indicative of moderate polydispersity. Thermogravimetric analysis (TGA, Figure 6D) showed that free G24 underwent a single‐stage thermal decomposition between 208 and 363 °C. In contrast, G24‐loaded PU‐MCs exhibited three distinct thermal degradation stages: initial moisture release (<100 °C), followed by urethane matrix decomposition (100–314 °C), and subsequent G24 volatilization (314–363 °C). This thermal behavior suggests an enhanced thermal stability of G24 upon encapsulation within the PU‐MCs. Finally, a linear relationship was observed between drug loading (LC) and encapsulation efficiency (EE) within the concentration range of 25–125 µg mL^−1^, yielding calculated values of 9.14% and 89.41%, respectively.

### 2.11 Surface Tension and Contact Angle

**Figure 8:**
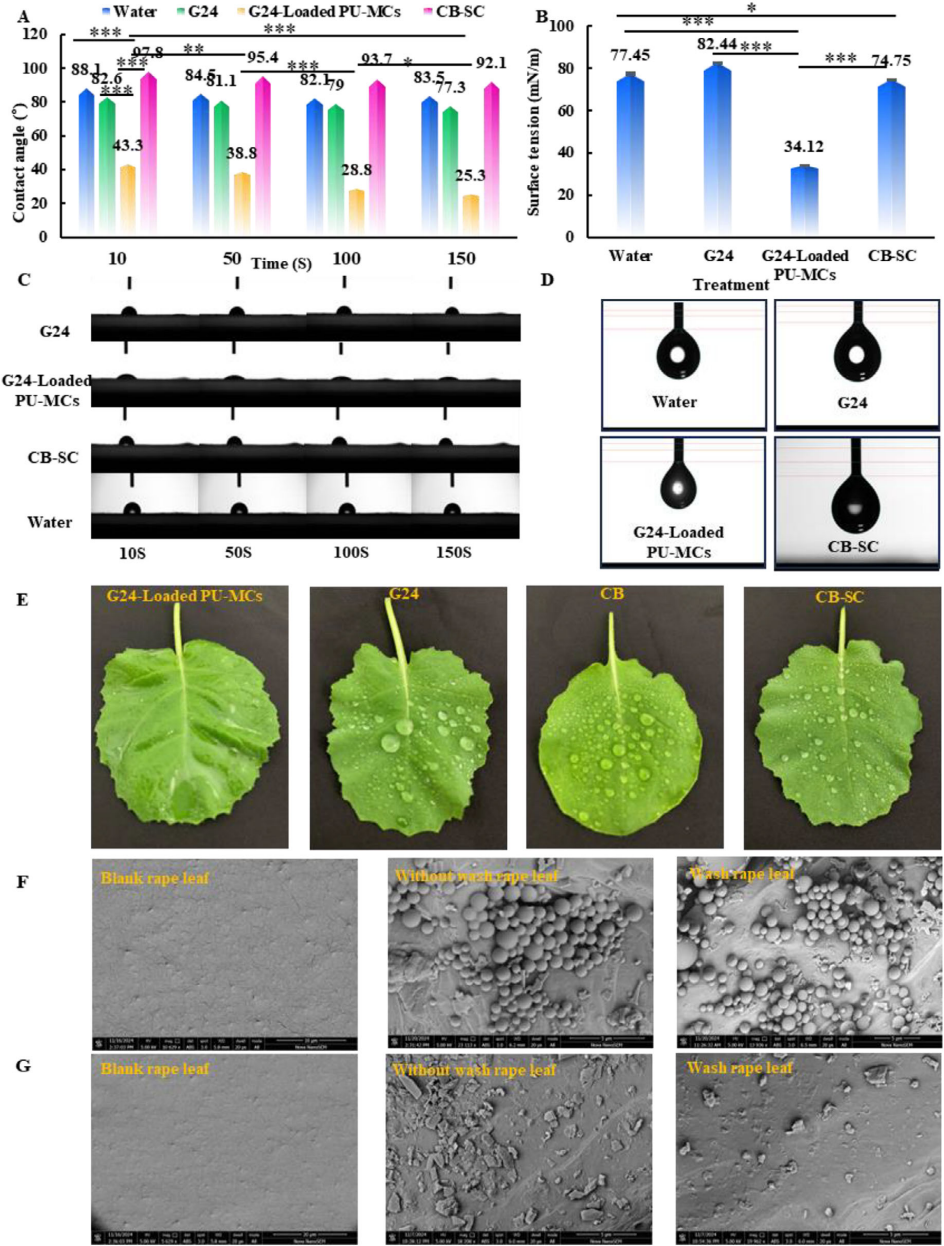
A) Contact angles for different solutions used to wet tender leaves for 10, 50, 100, 150 s; B) Surface tension data for different solutions; C) Images of contact angles for different solutions on tender leaves at 10, 50, 100, and 150 s. D) Images of surface tension; E)Wetting of rape leaves with different droplets; F) Scanning electron microscope (SEM) images showing adhesion of the G24‐loaded PU‐MCs on rape leaves without washing and after washing; G) Scanning electron microscope (SEM) images showing adhesion of the CB‐SC on rape leaves without washing, after washing. Statistically significant differences between the means were analyzed with one‐way ANOVA, followed by the least significant difference (LSD) post‐hoc test in (A, B) (For all studies, *n* = 3; ^*^ *p* < 0.05, ^**^ *p* < 0.01, ^***^ *p* < 0.001; n.s. = no significance).

The wetting characteristics of pesticide formulations significantly influence their deposition on crop leaves. In this study, we evaluated the wetting properties of nanoformulations by measuring the contact angles on rape leaves and the surface tensions of the droplets, as illustrated in **Figure** **8**B,D. The surface tensions recorded for water, a 100 mg L^−1^ G24 solution, a 100 mg L^−1^ G24‐Loaded PU‐MCs solution, and a 100 mg L^−1^ CB‐SC solution were 77.45, 82.44, 34.12, and 74.75 mN m^−1^, respectively. Notably, the surface tension of the G24‐Loaded PU‐MCs was significantly lower than that of the CB‐SC formulation.

The contact angles of water, G24, and the carbendazim‐SC solution on the surface of rape leaves exhibited minimal variation, measuring 83.5°, 77.3°, and 92.1° at 150 s, respectively (Figure 8A,C). In contrast, the contact angles of the G24‐Loaded PU‐MCs solutions demonstrated considerable variation; the initial contact angle was 43.3° at 10 s, decreasing to 25.3° at 150 s. These results indicate that G24‐Loaded PU‐MCs exhibit superior wetting properties, facilitating effective deposition on rape leaves. This pronounced temporal change highlights the remarkably rapid‐wetting capability of G24‐Loaded PU‐MCs, which likely promotes uniform droplet spreading and enhances foliar deposition efficiency (Figure 8E).

### 2.12 Release Behavior of G24‐Loaded PU‐MCs

The release characteristics of G24 from the MCs were thoroughly evaluated through longitudinal monitoring of cumulative release profiles (Figure 7A,B). The results demonstrated biphasic release kinetics: an initial burst release phase (0–3 h) characterized by a rapid dissolution of drug molecules situated at or near the microcapsule surface, followed by a sustained diffusion‐controlled release phase (> 3 h). The initial burst, which is crucial for achieving immediate therapeutic drug concentrations at target sites, exhibited a steep release rate. Subsequently, the release profile transitioned to a slower, steady phase, primarily governed by drug permeation through the internal pores of the microcapsules. This mechanism is conducive to maintaining prolonged effective plasma concentrations. These findings provide essential theoretical support for the optimization of controlled‐release performance in microcapsule‐based drug delivery systems.

### 2.13 Analysis of the Retention and Adhesive Properties of G24‐Loaded PU‐MCs on Rape Leaves

The deposition behavior and adhesion properties of G24‐loaded PU‐MCs were systematically investigated on rapeseed (*Brassica napus L*.) leaves to assess their potential for agricultural applications (Figure 7D). Quantitative analysis of leaf retention showed values of 6.93, 17.13, 8.60, and 6.67 mg cm^−^^2^ for a 100 mg L^−1^ solution of G24, G24‐loaded PU‐MCs, CB‐SC, and CB, respectively. A simulated rainfall erosion assay (45° inclination) revealed that 74.3% of both G24‐loaded PU‐MCs and free G24 remained adhered to the leaf surface following deionized water washing, while minimal retention was observed for the CB‐SC formulation (Figure 8F,G). Microencapsulation resulted in an approximately two‐fold increase in leaf adhesion compared to conventional formulations. These results indicate that G24‐loaded PU‐MCs significantly enhance field application efficiency and rainfastness, highlighting a promising approach for optimizing foliar delivery efficacy in crop protection strategies.

### 2.14 Safety Assessment of G24‐Loaded PU‐MCs to Rapeseed Plants

**Figure 9:**
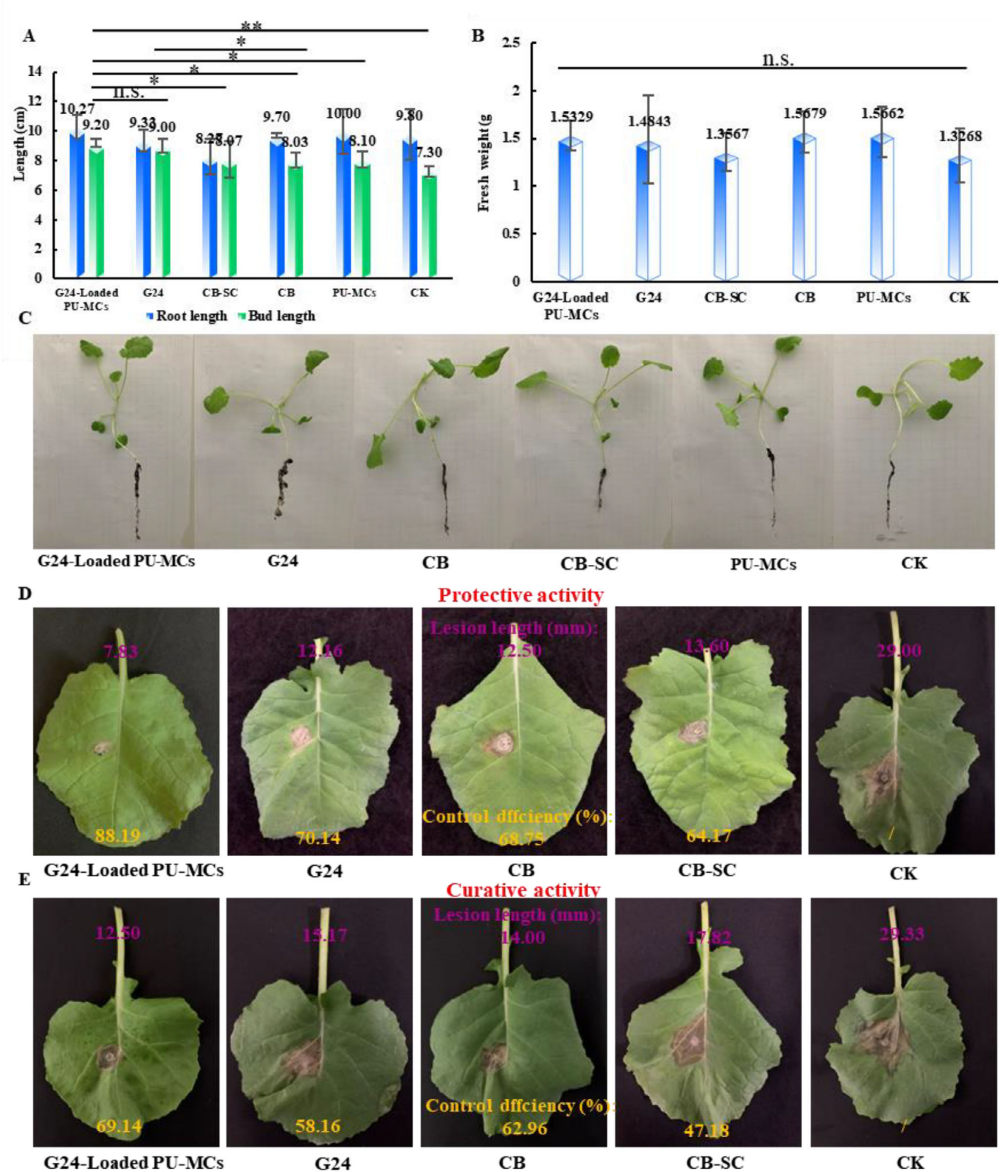
A,B) Data graphs and C) digital photos of the root length, bud length, and fresh weight of the oilseed rape after spraying with 200 mg mL^−1^ of G24‐loaded polyurethane microcapsules (G24‐Loaded PU‐MCs), G24, CB (Carbendazim), CB‐SC (Carbendazim Suspension Concentrates), and water for 7 days; D,E) in vivo the control efficacy of the G24‐Loaded PU‐MCs, G24, CB, CB‐SC, water against *S. sclerotiorum* on the oilseed rape. Statistically significant differences between the means were analyzed with one‐way ANOVA, followed by the least significant difference (LSD) post‐hoc test in (A) (For all studies, *n* = 10; ^*^ *p* < 0.05, ^**^ *p* < 0.01, ^***^ *p* < 0.001; n.s. = no significance).

The growth response of rapeseed seedlings provides robust evidence supporting the safe agricultural application of G24‐Loaded PU‐MCs. A comprehensive evaluation of phenotypic development parameters, including root length, shoot length, and fresh weight, was conducted across treatment groups (G24 solution, G24‐PU‐MCs, empty PU‐MCs solutions, CB, CB‐SC, and water) over a 7 day exposure period. The G24‐Loaded PU‐MCs treatment group exhibited morphological parameters–root length (9.2 ± 0.3 cm), shoot length (10.3 ± 0.8 cm), and fresh weight (1.5329 ± 0.16 g), that were statistically indistinguishable from those of the control group (**Figure** **9**A–C). Notably, no significant growth inhibition was observed, even at a high concentration of 200 mg L^−1^, demonstrating the exceptional biocompatibility of the PU‐MCs. The preservation of normal physiological development patterns under this material treatment highlights its potential as an environmentally sustainable agricultural enhancer, particularly relevant for sustainable farming systems requiring controlled and sustained release of growth regulators.

## 3 Conclusion

This study reports the rational design and synthesis of novel chromone derivatives incorporating an acylhydrazone moiety, specifically targeting *S. sclerotiorum*, a major fungal pathogen. Compound G24 exhibited potent antifungal activity, likely attributed to its dual mechanism of action involving disruption of fungal hyphal morphology and destabilization of intracellular microtubule architecture. In planta assays revealed that G24 conferred significant therapeutic (69.14%) and prophylactic (88.19%) effects against *S. sclerotiorum* at a concentration of 100 µg mL^−1^. Furthermore, G24 displayed favorable pharmacokinetic properties and low cytotoxicity. To address formulation limitations, G24 was encapsulated within polyurethane microcapsules (PU MCs) via interfacial polymerization, achieving controlled release kinetics. In pot trials, G24‐loaded PU MCs outperformed the commercial formulation CB‐SC, demonstrating a 24.02% increase in protective efficacy and a 21.96% improvement in curative activity, highlighting their potential as environmentally sustainable carriers. Previously reported methods (primarily biocontrol) emphasize sustainability, managing disease through microbes and molecular insights, but their field efficacy is often environment‐dependent and less stable. In contrast, the newly developed compound G24 and its microencapsulated formulation demonstrate significantly superior targeted control efficacy over conventional fungicides. G24 targets fungal tubulin polymerization, exhibits strong selectivity, and is safe for mammals. Microencapsulation enhances field persistence, adhesion, and slow‐release kinetics, offering a stable, highly effective, and innovatively‐mechanized solution that addresses pathogen resistance challenges. These results not only advance the structural innovation of tubulin‐targeting antifungals but also exemplify a synergistic approach combining molecular design and formulation engineering to enhance agrochemical sustainability. Moving forward, we will accelerate the application process in agriculture through field experiments and optimization of microcapsule technology.

## 4 Experimental Section

### General Information

All chemical reagents and solvents employed in this investigation were analytical‐grade materials. Nuclear Magnetic Resonance (NMR) spectral data were acquired using a JEOL ECS‐500 MHz spectrometer (JEOL, Japan). Mass spectrometric analyses were conducted on a Thermo Scientific Q Exactive series chromatographic system equipped with an electrospray ionization (ESI) interface. Melting point determinations were performed with an X‐4D precision melting point apparatus. Fungal cellular membrane ultrastructure was examined through scanning electron microscopy using a FEI NovaNano SEM 450 system (FEI, USA). Tubulin fluorescence dynamics in *S. sclerotiorum* were visualized and analyzed under an Olympus BX53 fluorescence microscope (Olympus, Japan). Fourier‐transform infrared (FTIR) spectroscopic measurements were carried out using a Thermo Fisher Scientific spectrometer (USA). Nanoparticle size distribution profiles were determined through dynamic light scattering analysis employing a Zetasizer Nano ZS90 instrument (Malvern Instruments, UK). The isolate used in this study was obtained from [*Sclerotinia sclerotiorum* (*S. sclerotiorum*, BNCC122299) was gained from the BeiNa Culture Collection, the carbendazim‐resistant strains (*Sclerotinia sclerotiorum*) were provided by the College of Plant Protection, Nanjing Agricultural University].

### Preparation of Target Compounds H

**Scheme 1:**
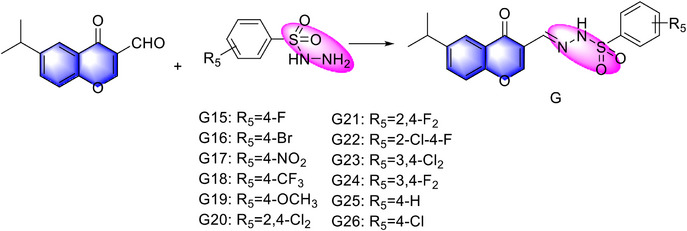
Synthetic route of the series compounds **G**.

A mixture of compound A (1.0 mmol) dissolved in anhydrous ethanol (15 mL) was charged with substituted benzoylhydrazine derivative B (1.0 mmol) and glacial acetic acid (0.1 mmol) under ambient conditions. The reaction system was maintained under vigorous agitation at room‐temperature (25 ± 2 °C) for 6 h using a magnetic stirrer. Upon completion, the resultant precipitate was isolated through vacuum filtration and subsequently purified via recrystallization from ethanol to yield compound G as crystalline solids. Product identity was confirmed by comparative analysis with authentic spectral data (^1^H/^13^C NMR, HRMS) and melting point determination. (**Scheme** **1**).^[^ ^30^ ^]^

### Anti‐Fungal Activity In Vitro and In Vivo

The in vitro antifungal efficacy of target compound G was evaluated against six phytopathogenic fungal species using the mycelial linear growth inhibition method.^[^ ^31^, ^32^ ^]^ Test solutions of compound G were prepared at a concentration of 25 µg mL^−1^. Commercial broad‐spectrum antifungal agents carbendazim (CB) and its suspension concentrate formulation (CB‐SC) served as positive controls. Following in vitro evaluation, the protective and curative effects of derivative G24 against *S. sclerotiorum* were investigated in vivo. For protective efficacy assays, uniformly developed 30‐day‐old rapeseed (*Brassica napus*) leaves were selected. On day 1, leaves were treated by foliar spraying with G24 (100 µg mL^−1^), while control groups received distilled water. After 24 h, leaves were inoculated with 5 mm mycelial plugs of *S. sclerotiorum*. Treated plants were cultivated under controlled greenhouse conditions (21 °C, 85% relative humidity) for 72 h to promote pathogen development. Protective efficacy was calculated using the formula:

(1) $$\mathrm{Protective}\;\;\mathrm{efficacy}\left(\%\right)=\left[\left(D-T\right)/\left(D-5\right)\right]\times 100$$

where D and T represent lesion diameters (mm) in distilled water‐treated controls and G24‐treated samples, respectively. For curative effect evaluation, mycelial inoculation preceded antifungal treatment by 24 h, with subsequent steps identical to the protective protocol. All experiments were performed in two independent trials, each containing three biological replicates per treatment group to ensure statistical robustness.

### Fungal Sample Preparation and Scanning Electron Microscopy Analysis

Mycelial segments (5.0 × 4.0 mm) were excised from fungal colonies following treatment with G24 at concentrations of 50 and 25 µg mL^−1^, along with solvent control (0.5% dimethyl sulfoxide). Specimens were fixed by immersion in 2.5% glutaraldehyde solution at 4 °C for 16–18 h. Subsequent processing involved three sequential 15 min washes with 0.1 m phosphate buffer (pH 7.4). Dehydration was performed through a graded ethanol series (20%, 50%, 80%, and 100%), with each concentration maintained for 5 min under vacuum conditions. Prior to microscopic examination, samples were subjected to gold sputter coating using an ion sputter coater (JFC‐1100, JEOL) to achieve optimal surface conductivity. Morphological characterization was conducted via field emission scanning electron microscopy (FE‐SEM, JSM‐6700F, JEOL) operating at 5 kV accelerating voltage. All experimental procedures were performed in triplicate to ensure methodological reproducibility.^[^ ^7^, ^33^ ^]^

### Effects of Sclerotia Germination Inhibition

*S. sclerotiorum* was cultured for 21 days to produce sclerotia. A stock solution of compound G24, CB, and azoxystrobin were prepared and diluted to final concentrations of 25, 12.5, 6.25, 3.125, 1.5625, and 0.78125 µg mL^−1^. Nine sclerotia were then inoculated onto agar plates containing each concentration. Each treatment was replicated three times. Azoxystrobin and CB served as a positive control, while sterile distilled water was used as a negative control. All plates were incubated at 25 °C for 3 days, after which the inhibitory effect of G24 on sclerotial germination was determined.

### Microtubule Antibody Staining

The effect of Compound G24 on mycelial microtubules was investigated. Mycelial cultures were treated with Compound G24 at concentrations of 6.25 and 3.125 µg mL^−1^ and incubated at 25 °C for 24 h. Following incubation, mycelia were harvested, fixed, and stained with a microtubule‐specific antibody (0.5 µL). Samples were then incubated in the dark at 5 °C for 12 h, followed by three washes with ice‐cold 0.01 M PBS (5 min each). Mycelia were mounted on sterile slides, counterstained with DAPI to quench background fluorescence, and visualized using fluorescence microscopy.^[^ ^34^ ^]^

### Pharmacokinetic Prediction

The drug‐likeness of compound G24 was evaluated using absorption, distribution, metabolism, excretion, and toxicity (ADMET) properties predicted by the ADMETlab 2.0 web server (https://admetmesh.scbdd.com). ADMET prediction was a crucial step in drug discovery, providing insights into the pharmacokinetic and toxicological characteristics that influence a compounds ability to reach its intended site of action and exert its therapeutic effect.

### Molecular Docking

Molecular docking of tubulin with small molecules was conducted using AutoDock Vina version 1.2.3. The protein sequences for *α*‐tubulin and *β*‐tubulin from *S. sclerotiorum* were obtained from the National Center for Biotechnology Information (NCBI) protein repository. The tubulin model was constructed using AlphaFold version 2.3, employing the “monomer” AI model to explore all genetic databases utilized in CASP14. Subsequently, the Amber force field in OpenMM was applied to relax the protein structure, and the model with the highest confidence was selected as the experimental model based on the pLDDT score ranking.

Pre‐docking preparation was performed using AutoDock Tools, where non‐polar hydrogen atoms were merged, and Gasteiger charges were assigned to generate the receptor PDBQT file. The small molecule ligands underwent a similar preparation process; their PDB files were converted to PDBQT format using AutoDock Tools, ensuring appropriate assignment of rotatable bonds and partial charges. The docking simulation was executed with the command vina –config config.txt –log log.txt –out out.pdbqt, where the configuration file specified the PDBQT files for both the receptor and ligand, as well as the grid center, size, and other docking parameters. The output PDBQT file contained the predicted binding poses, which were subsequently analyzed to identify the most favorable binding interactions. Visualization and further analysis of the docking results were conducted using PyMOL and Discovery Studio 2020.

### Molecular Dynamics (MD)

Molecular dynamics (MD) simulations were performed using GROMACS 2022.3. The small molecule was parameterized using the GAFF force field implemented in AmberTools22. Partial atomic charges were derived using Gaussian16W calculations followed by RESP fitting. These charges were then incorporated into the system's topology file. The MD system was solvated with TIP3P water molecules and neutralized by the addition of Na+ ions. The Amber99sb‐ildn force field was employed for the protein. The system was energy‐minimized using the steepest descent algorithm. Following energy minimization, the system was equilibrated using 100 000 steps each of NVT and NPT ensembles, with a coupling constant of 0.1 ps for 100 ps. A production MD simulation was then conducted for 5 000 000 steps with a 2 fs time step, resulting in a total simulation time of 100 ns. Trajectory analysis was performed using GROMACS built‐in tools to calculate root‐mean‐square deviation (RMSD), root‐mean‐square fluctuation (RMSF), and free energy landscapes based on the atomic trajectories of each amino acid residue.

### Cytotoxicity Assay

Cytotoxicity assays against NRK and SV‐HUC‐1 cell lines were conducted using the MTT method, as previously described. The cell lines were exposed to varying concentrations of the target compound G24 (100, 50, 25, 12.5, and 6.25 µg mL^−1^) in growth medium for a duration of 48 h. Absorbance was measured at 490 nm, with five replicates performed for each concentration.

### Preparation of Empty PU‐MCs and G24‐Loaded PU‐MCs

G24‐loaded polyurethane microcapsules (PU‐MCs) were prepared as follows: Initially, 6.67 g of isophorone isocyanate, 1.02 g of G24, and 35 g of 150# solvent oil were added to 33 g of an EL‐40 aqueous solution and subjected to high‐shear mixing. Subsequently, a mixture of A‐95, 1.30 g of pentaerythritol, and 10 g of water was added and reacted for 2 h. Following this, 0.51 g of XC 258 wetting agent was incorporated. Finally, 6.12 g of a xanthan gum aqueous solution (thickener) and 4.08 g of 811 dispersant were added to the mixture to obtain the G24‐loaded PU‐MCs.^[^ ^35^ ^]^

### Characterization of G24‐Loaded PU‐MCs

The initial morphology of the G24‐loaded PU‐MCs was observed via optical microscopy (Ti‐S). Surface characteristics were further investigated using scanning electron microscopy (SEM) with a FEI instrument (USA). The average particle size of the G24‐loaded PU‐MCs was measured at room‐temperature by dynamic light scattering (DLS) using a Malvern Zetasizer Nano‐ZS instrument (Nano Brook Omni, Brookhaven). Chemical composition was analyzed by Fourier transform infrared (FTIR) spectroscopy using a PerkinElmer spectrometer (UK) across a wavenumber range of 500–4000 cm^−1^. Thermal stability was evaluated by thermogravimetric analysis (TGA) using a NETZSCH STA 449F5 thermal analyzer (Germany). Samples were heated from 30 to 800 °C at a rate of 10 °C min^−1^ under a nitrogen atmosphere (flow rate: 50 mL min^−1^). The G24‐loaded PU‐MCs were washed three times and dried before undergoing the aforementioned characterization procedures.^[^ ^35^ ^]^

### The Loading Content (LC) and the Encapsulation Efficiency (EE) Determination of the G24‐Loaded PU‐MCs were Measured

A total of 4 mL of G24‐loaded polyurethane microspheres (PU‐MCs) was subjected to centrifugation at 5000 rpm for 5 min. Following centrifugation, 1 mL of the supernatant was transferred to a dark volumetric flask and diluted to a final volume of 25 mL with a methanol‐water solution (7:3, v/v). The solution was then filtered through a 0.24 µm pore size filter. The concentration of free G24 in the supernatant was subsequently analyzed using high‐performance liquid chromatography (HPLC).^[^ ^36^ ^]^

The HPLC analysis was conducted under the following conditions: an Agilent XDB‐C18 reverse phase column (5 µm, 4.6 × 250 mm) was utilized, with the column maintained at a temperature of 35 °C. The mobile phase consisted of a methanol‐water solution (7:3, v/v) at a flow rate of 1 mL min^−1^. Detection was performed at a wavelength of 310 nm, and the injection volume was set at 5 µL. The loading capacity (LC) and encapsulation efficiency (EE) of G24 were calculated using the appropriate equations:

(2) $$\mathrm{LC}\%=\frac{m_{I}-m_{i}}{m_{I}-m_{i}+m_{0}}\times 100\%$$

(3) $$\mathrm{EE}\%=\frac{m_{I}-m_{i}}{m_{I}}\times 100\%$$

*m_I_* is the weight of the total G24 added, *m_i_* is the weight of free G24 in the supernatant, and *m_O_* is the weight of the blank capsule.

### Surface Tension and Contact Angle

The contact angles of deionized water, 100 mg L^−1^ CB‐SC, 100 mg L^−1^ G24, and 100 mg L^−1^ G24‐loaded PU‐MCs were evaluated on rape leaves using a contact angle meter (SINDIN, SDC‐350, China) at a controlled temperature of 25 °C. For each measurement, a 10 µL aliquot of the respective solution was carefully dispensed onto the leaf surface via a microsyringe. Contact angle measurements were then acquired at pre‐determined time intervals of 10, 50, 100, and 150 s. Surface tension measurements were conducted using the pendant drop technique. All experiments were replicated five times (*n* = 5).^[^ ^37^ ^]^

### Release Behavior of G24‐Loaded PU‐MCs

Approximately 10 mL of G24‐loaded PU‐MCs (40 mg L^−1^) were placed in a dialysis bag, which was then immersed in 50 mL of a methanol/water solution (7:3, v/v). At predetermined time intervals, 1 mL aliquots were withdrawn and filtered through a 0.22 µm membrane filter. The filtrate was analyzed by HPLC using a previously established calibration curve. To maintain a constant volume, 1 mL of fresh methanol/water solution (7:3, v/v) was added to the dialysis medium after each sampling. The release ratio of G24 from PU‐MCs was subsequently calculated:

(4) $$cumulative\;release\;percentage=\sum \frac{M_{t}}{M_{o}}\times 100\%$$

where *M_t_* is the cumulative amount of G24 released at the corresponding sampling time point, M0 is the initial weight of the G24 load in the particle, and t is the sampling time.

### Analysis of the Retention and Adhesive Properties of G24‐Loaded PU‐MCs on Rape Leaves

Leaf segments (2 cm^2^) were initially immersed in test solutions (water, 100 mg L^−1^ CB‐SC, 100 mg L^−1^ G24, and 100 mg L^−1^ G24‐loaded PU‐MCs) for 30 s, followed by removal. The retention of on the leaf surface was determined by quantifying the mass change of the test solution.^[^ ^38^ ^]^

(5) $$R_{m}=\frac{W_{1}-W_{0}}{S}$$

where *W_0_* (mg) signifies the leaf weight before soaking, *W_1_* (mg) signifies the leaf weight after soaking, and *S* signifies the blade area.

Subsequently, a custom‐built rainwater simulation device was employed to assess the adhesion of materials. Leaves were positioned on a glass plate inclined at 45° and subjected to five simulated rainfall events, each consisting of 1 mL of water, with complete drying between each event. Following the simulated rainfall, the following analyses were performed: 1) The morphology of CB‐SC and G24‐loaded PU‐MCs adhering to the leaf surface was characterized using Scanning Electron Microscopy (SEM); 2) Leaves were placed in centrifugal tubes with 7 mL of methanol, centrifuged at 8000 rpm for 10 min, and the resulting supernatant was analyzed to determine the concentration of G24. The residual rate (N) of G24 was then calculated using the following equation:

(6) $$N=\frac{A_{0}}{A_{1}}\times 100$$

where A_0_ signifies the G24 content after scouring and A_1_ signifies the controls.

### Safety Assessment of G24‐Loaded PU‐MCs to Rapeseed Plants

To evaluate the biosafety of G24‐loaded PU‐MCs on crop plants, the growth parameters of oilseed rape (*Brassica napus*) seedlings were assessed. 2‐week‐old seedlings were foliar‐sprayed with aqueous suspensions of the following treatments (200 mg L^−1^): control (water), CB, CB‐SC, G24, and G24‐loaded PU‐MCs. Following spraying, plants were air‐dried at ambient temperature and the rape seedlings were placed in a constant temperature and humidity incubator for one week (25 °C, 80% humidity, and a light:dark ratio of 16:8). After 7 days, fresh weight, root length, and stem length were measured for each treatment. Each treatment group had 10 plants and the process was repeated three times.^[^ ^39^ ^]^

### Statistical Analysis

The SPSS 23.0 statistical analysis software was applied for statistical analysis, statistically significant differences between the means were analyzed with one‐way ANOVA, followed by the least significant difference (LSD) post‐hoc test (For all studies, n ≥ 3; ^*^ *p* < 0.05, ^**^ *p* < 0.01, ^***^ *p* < 0.001; n.s. = no significance). Microsoft Office Excel 2023 and Origin 2025 were used for data fitting analysis. Data were expressed as the mean ± standard error of the mean for all the experiments.

### Compliance with Ethics Requirements

This article does not contain any studies with human or animal subjects.

## Conflict of Interest

The authors declare no conflict of interest.

## Author Contributions

L.S. contributes to writing (review & editing and original draft), supervision, project administration, methodology, investigation, data curation, and conceptualization; X.H. and Y.W. perform formal analysis and data curation; X.Z. and B.Z. contribute to writing (review & editing) and funding acquisition; S.Y. contributes to writing (review & editing), supervision, resources, project administration, investigation, and funding acquisition.

## Supporting information



Supporting Information

## Data Availability

The data that support the findings of this study are available in the supplementary material of this article.
