# Supplementary material for: Novel Antifungal Scaffold Targeting Tubulin Overcomes Sclerotinia Sclerotiorum Resistance
Source: Adv Sci (Weinh). 2025 Nov 21;13(8):e11492. doi: 10.1002/advs.202511492 (PMC12884739; doi:10.1002/advs.202511492)
Supplement: Supplementary file 1 — Supporting Information [file ADVS-13-e11492-s001.docx]

**Supporting Information**

**Novel Antifungal Scaffold Targeting Tubulin Overcomes *Sclerotinia sclerotiorum* Resistance**

Lihui Shao^1,2^, Xianqun Hu^1^, Ying Wu^1^, Xiang Zhou^1*^, Bo Zhang^3*^, Song Yang ^1,2,*^

^1^ State Key Laboratory of Green Pesticide, Key Laboratory of Green Pesticide and Agricultural Bioengineering, Ministry of Education, Center for R&D of Fine Chemicals of Guizhou University, Guiyang, Guizhou 550025, People’s Republic of China.

^2^ School of Chemistry and Chemical engineering, Guizhou University, Guiyang, Guizhou 550025, People’s Republic of China

^3^ Shanghai Engineering Research Center of Green Energy Chemical Engineering, College of Chemistry and Materials Science, Shanghai Normal University, 100 Guilin Road, Shanghai, 200234, P. R. China.

^*^ Corresponding author.

Email: jhzx.msm@gmail.com or syang@gzu.edu.cn (S. Yang);

zb830216@shnu.edu.cn (B. Zhang);

xiangzhou@gzu.edu.cn or zhoux1534@163.com (X. Zhou).


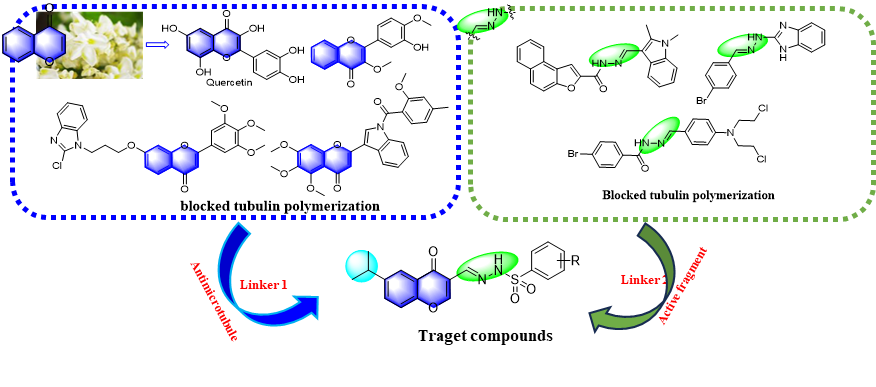


**Figure 1S.** Design of target compounds.

**Figure 2S.** The model of **G24** docking with mammalian tubulin molecules

**Table S1** EC_50_ values of compound **G** against *S. sclerotiorum*

| **Compounds** | *S. sclerotiorum* | | |
| --- | --- | --- | --- |
|  | **EC_50_ (µg/mL)** | **Regression Equation** | **R^2^** |
| **G15** | 2.4±0.5 | y=0.701x+4.735 | 0.982 |
| **G16** | 1.8±0.4 | y=0.640x+4.829 | 0.983 |
| **G17** | 1.9±0.5 | y=0.854x+4.767 | 0.988 |
| **G18** | 0.80±0.05 | y=0.644x+5.062 | 0.988 |
| **G19** | 0.91±0.03 | y=0.697x+5.028 | 0.989 |
| **G20** | 1.1±0. 2 | y=0.505x+4.970 | 0.981 |
| **G21** | 0.87±0.04 | y=0.652x+5.038 | 0.991 |
| **G22** | 0.69±0.07 | y=0.815x+5.131 | 0.992 |
| **G23** | 0.71±0.07 | y=0.479x+5.070 | 0.980 |
| **G24** | **0.21±0.04** | y=0.277x+5.187 | 0.987 |
| **G25** | 1.0±0.1 | y=0.639x+4.987 | 0.993 |
| **G26** | 0.79±0.3 | y=0.507x+5.052 | 0.999 |
| **CB** | 0.37±0.05 | y=0.482x+5.070 | 0.963 |
| **CB-SC** | 0.71±0.07 | y=0.481x+6.270 | 0.984 |
| **Azoxystrobin** | 0.36±0.05 | y=0.677x+5.294 | 0.949 |

**Table S2** Compound G24 tested for antifungal activity at 25 µg/mL (%) *in vitro*

| **Compd.** | ***G. z.*** | ***F. o.*** | | ***A. a.*** | ***V. dahlia*** | ***B. d.*** |
| --- | --- | --- | --- | --- | --- | --- |
| **G24** | 48.5±0.6 | 43.8±0.3 | 59.7±0.6 | | 58.9±0.4 | 49.3±0.5 |
| **CB** | 49.4±0.5 | 68.6±1.9 | 97.1±0.5 | | 72.4±1.1 | 52.1±1.6 |

*G. z., Gibberella zeae; A. a,Alternaria alternate; B. d., Botryosphaeria dothidea; F. o., Fusariumoxysporum; V. d., Verticillium dahlia*

**Table S3** Protective and curative Activities of G24-Loaded PU-MCs, G24, CB and CB-SC against *S. sclerotiorum* at 100*µ*g/mL *in vivo*

| **Compd.** | **Curative effect** | | **Protective effect** | |
| --- | --- | --- | --- | --- |
|  | **area per lesion**  **(mm ± SD) ^a^** | **Control efficiency (%)^a^** | **area per lesion**  **(mm ± SD) ^a^** | **Control efficiency (%) ^a^** |
| **G24** | 15.17±0.94^a^ | 58.16b | 12.16±0.79 | 70.14b |
| **G24-Loaded PU-MCs** | 12.50±0.72 | 69.14a | 7.83±0.45 | 88.19a |
| **CB** | 14.00±0.56 | 62.96b | 12.50±0.81 | 68.75b |
| **CB-SC** | 17.82±0.72 | 47.18c | 13.60±0.65 | 64.17c |
| **CK** | 29.33±0.78 | / | 29.00±1.03 | / |

Different lowercase letters indicate statistically significant differences (For all studies, n =10, *p* < 0.05), as determined by one-way ANOVA followed by Waller-Duncan’s post hoc test.

**Table S4.** ADMET properties and drug properties of compound G24 were predicted by AD-METab.

| **Category** | **Model** | **Value** | **Decision** |
| --- | --- | --- | --- |
| **Medicinal chemistry** | QED | 0.519 | Medium |
|  | SAscore | 2.0 | Excellent |
|  | NPscore | -1.429 | Excellent |
|  | Lipinski Rule | - | Excellent |
|  | Pfizer Rule | - | Excellent |
|  | GoldenT riangle | - | Excellent |
| **Absorption** | Caco-2 Permeability | -5.067 | Excellent |
|  | Pgp-inhibitor | 0.18 | Excellent |
|  | Pgp-substrate | 0.016 | Excellent |
|  | HIA | 0 | Excellent |
|  | F20% | 0.048 | Excellent |
|  | F30% | 0.003 | Excellent |
|  | F50% | 0.013 | Excellent |
| **Distribution** | PPB | 98.339% | Medium |
|  | OATP1B1 inhibitor | 0.215 | Excellent |
|  | BBB Penetration | 0 | Excellent |
|  | BCRP inhibitor | 0 | Excellent |
| **Metabolism** | CYP1A2 substrate | 0 | Excellent |
|  | CYP2D6 inhibitor | 0 | Excellent |
|  | CYP2C9 inhibitor | 0.234 | Excellent |
|  | CYP2C19 substrate | 0.095 | Excellent |
|  | CYP3A4 inhibitor | 0.504 | Medium |
|  | CYP2B6 substrate | 0 | Excellent |
| **Excretion** | CL | 4.676 | Excellent |
|  | T 1/2 | 0.731 | Medium |
| **Toxicity** | hERG Blockers | 0.01 | Excellent |
|  | A549 Cytotoxicity | 0 | Excellent |
|  | AMES Toxicity | 0.008 | Excellent |
|  | Rat Oral Acute Toxicity | 0.195 | Excellent |
|  | FDAMDD | 0.384 | Medium |
|  | Skin Sensitization | 0.023 | Excellent |
|  | Eye Corrosion | 0 | Excellent |
|  | Eye Irritation | 0.068 | Excellent |
|  | Respiratory Toxicity | 0.003 | Excellent |

**Table S5** *In vitro* cytotoxicity of compound **G24** against NRK and SV-HUC-1.

| Compound | NRK | | SV-HUC-1 | |
| --- | --- | --- | --- | --- |
|  | Concentration (µmol/L) | inhibition (%) | Concentration (µmol/L) | inhibition (%) |
| **G24** | 100 | 47.90±1.99 | 100 | 27.59±2.43 |
|  | 50 | 38.94±1.21 | 50 | 8.66±1.35 |
|  | 25 | 14.87±1.85 | 25 | 0 |
|  | 12.5 | 0 | 12.5 | 0 |
|  | 6.25 | 0 | 6.25 | 0 |
| 5-Fluorouracil | 100 | 49.40±1.25 | 100 | 57.17±0.56 |
|  | 50 | 41.89±1.19 | 50 | 39.44±3.01 |
|  | 25 | 30.05±0.28 | 25 | 20.83±2.13 |
|  | 12.5 | 16.16±1.15 | 12.5 | 12.13±3.65 |
|  | 6.25 | 4.24±0.27 | 6.25 | 11.59±0.51 |

**Table S6** Compound **G24** tested for Carbendazim-resistant strain (*C-S. sclerotiorum*) at 25 µg/mL (%) *in vitro*.

| **Compd.** | ***C-S. sclerotiorum*** |
| --- | --- |
| **G24** | 28.5±0.6 |
| **CB** | 6.4±0.4 |

**^1^H NMR, ^13^C NMR and HRMS spectra data for target compounds.**

**(*E*)-4-fluoro-*N'*-((6-isopropyl-4-oxo-4*H*-chromen-3-yl)methylene)benzenesulfonohydrazide (G15)**

White solid, m.p.183−184°C, yield 62%; ^1^H NMR (500 MHz, DMSO-d6) δ 9.84 (s, 1H), 7.40 (s, 1H), 6.93 (s, 1H), 6.82 (d, J = 7.0 Hz, 2H), 6.80 (d, J = 1.7 Hz, 1H), 6.69 (d, J = 7.0 Hz, 1H), 6.65 (d, J = 6.9 Hz, 2H), 6.58 (d, J = 7.0 Hz, 1H), 2.93 (hept, J = 5.5 Hz, 1H), 1.47 (d, J = 6.9 Hz, 6H); ^13^C NMR (101 MHz, DMSO-d6) δ 147.7, 131.4, 131.2, 125.0, 120.2, 118.3, 118.0, 114.6, 111.5, 111.2, 106.3, 105.1, 102.8, 101.9, 34.2, 26.8; HRMS (ESI) m/z [M+H]^+^calcd for C_19_H_18_N_2_O_4_FS: 389.0966, found: 389.0950.

**(*E*)-4-bromo-*N'*-((6-isopropyl-4-oxo-4*H*-chromen-3-yl)methylene)benzenesulfonohydrazide (G16)**

yellow solid, m.p. 223−224°C, yield 63%; ^1^H NMR (500 MHz, DMSO-d6) δ 11.64 (s, 1H), 8.59 (s, 1H), 8.00 (s, 1H), 7.84 (d, J = 2.3 Hz, 1H), 7.79 (s, 4H), 7.70 (d, J = 8.7 Hz, 1H), 7.57 (d, J = 8.7 Hz, 1H), 3.00 (hept, J = 6.8 Hz, 1H), 1.18 (d, J = 6.9 Hz, 6H); ^13^C NMR (101 MHz, DMSO-d6) δ 147.7, 131.5, 131.2, 125.1, 120.2, 118.3, 114.6, 113.8, 111.3, 109.6, 106.3, 105.2, 102.8, 101.9, 34.2, 26.8; HRMS (ESI) m/z [M+H]^+^calcd for C_19_H_18_N_2_O_4_BrS: 449.0165, found: 449.0143.

**(*E*)-*N'*-((6-isopropyl-4-oxo-4*H*-chromen-3-yl)methylene)-4-nitrobenzenesulfonohydrazide (G17)**

White solid, m.p. 166−167°C, yield 61%; ^1^H NMR (500 MHz, DMSO-d_6_) δ 11.90 (s, 1H), 8.62 (s, 1H), 8.38 (d, J = 8.9 Hz, 2H), 8.12 (d, J = 8.9 Hz, 2H), 8.02 (s, 1H), 7.83 (d, J = 2.3 Hz, 1H), 7.70 (d, J = 8.7 Hz, 1H), 7.57 (d, J = 8.7 Hz, 1H), 3.00 (hept, J = 6.9 Hz, 1H), 1.18 (d, J = 6.9 Hz, 6H); ^13^C NMR (101 MHz, DMSO-d_6_) δ 175.2, 155.1, 154.7, 150.4, 147.0, 144.6, 141.5, 134.0, 129.3, 125.1, 123.5, 122.1, 119.2, 117.9, 33.4, 24.1; HRMS (ESI) m/z [M+H]^+^calcd for C_19_H_18_N_3_O_6_S: 416.0911, found: 416.0891.

**(*E*)-*N'*-((6-isopropyl-4-oxo-4*H*-chromen-3-yl)methylene)-4-(trifluoromethyl)benzenesulfonohydrazide (G18)**

White solid, m.p.207−208°C, yield 57%; ^1^H NMR (500 MHz, DMSO-d_6_) δ 11.81 (s, 1H), 8.61 (s, 1H), 8.09 (s, 1H), 8.07 (s, 1H), 8.01 (d, *J* = 0.4 Hz, 1H), 7.97 (d, *J* = 8.4 Hz, 2H), 7.83 (d, *J* = 2.3 Hz, 1H), 7.70 (d, *J* = 8.7 Hz, 1H), 7.57 (d, *J* = 8.7 Hz, 1H), 3.00 (hept, *J* = 6.9 Hz, 1H), 1.18 (d, *J* = 6.9 Hz, 6H); ^13^C NMR (101 MHz, DMSO-d_6_) δ 147.6, 131.5, 131.2, 125.1, 122.1, 120.5, 114.6, 110.5, 109.1, 109.1, 106.3, 105.2, 102.8, 101.8, 34.2, 26.8; HRMS (ESI) m/z [M+H]^+^calcd for C_20_H_18_N_2_O_4_F_3_S: 439.0934, found: 439.0925.

**(*E*)-*N'*-((6-isopropyl-4-oxo-4*H*-chromen-3-yl)methylene)-4-methoxybenzenesulfonohydrazide (G19)**

White solid, m.p. 145−146°C, yield 48%; ^1^H NMR (500 MHz, DMSO-d_6_) δ 11.39 (s, 1H), 8.55 (s, 1H), 7.97 (s, 1H), 7.84 (d, *J* = 2.3 Hz, 1H), 7.79 (s, 1H), 7.78 (d, *J* = 2.1 Hz, 1H), 7.70 (d, *J* = 8.7 Hz, 1H), 7.57 (d, *J* = 8.7 Hz, 1H), 7.09 (d, *J* = 2.1 Hz, 1H), 7.07 (d, *J* = 2.1 Hz, 1H), 3.78 (s, 3H), 3.00 (hept, *J* = 6.9 Hz, 1H), 1.19 (d, *J* = 6.9 Hz, 6H); ^13^C NMR (101 MHz, DMSO-d_6_) δ 147.8, 138.1, 131.43, 131.4, 125.2, 119.6, 114.8, 112.4, 111.6, 106.4, 105.3, 103.0, 102.2, 99.6, 52.5, 34.4, 27.0; HRMS (ESI) m/z [M+H]^+^calcd for C_20_H_21_N_2_O_5_S: 401.1166, found: 401.1147.

**(*E*)-2,4-dichloro-*N'*-((6-isopropyl-4-oxo-4*H*-chromen-3-yl)methylene)benzenesulfonohydrazide (G20)**

White solid, m.p.217−218°C, yield 66%; ^1^H NMR (500 MHz, DMSO-d_6_) δ 12.17 (s, 1H), 8.41 (s, 1H), 8.14 (s, 1H), 8.01 (d, *J* = 8.6 Hz, 1H), 7.85 (d, *J* = 2.1 Hz, 1H), 7.84 (d, *J* = 2.3 Hz, 1H), 7.69 (d, *J* = 8.7 Hz, 1H), 7.64 (d, *J* = 8.6 Hz, 1H), 7.54 (d, *J* = 8.7 Hz, 1H), 3.00 (hept, *J* = 6.9 Hz, 1H), 1.18 (d, *J* = 6.9 Hz, 6H); ^13^C NMR (101 MHz, DMSO-d_6_) δ 147.7, 131.3, 131.2, 125.0, 119.8, 118.9, 116.2, 114.6, 114.2, 113.4, 113.0, 110.4, 106.3, 105.1, 102.8, 101.8, 34.2, 26.8; HRMS (ESI) m/z [M+H]^+^calcd for C_19_H_17_N_2_O_4_Cl_2_S: 439.0281, found: 439.0259.

**(*E*)-2,4-difluoro-*N'*-((6-isopropyl-4-oxo-4*H*-chromen-3-yl)methylene)benzenesulfonohydrazide (G21)**

White solid, m.p.183−184°C, yield 69%; ^1^H NMR (500 MHz, DMSO-d_6_) δ 11.49 (s, 1H), 8.57 (d, *J* = 0.6 Hz, 1H), 7.97 (d, *J* = 0.6 Hz, 1H), 7.83 (d, *J* = 2.3 Hz, 1H), 7.75 – 7.68 (m, 3H), 7.57 (d, *J* = 8.7 Hz, 1H), 7.37 (dd, *J* = 8.6, 0.6 Hz, 2H), 3.01 (dq, *J* = 13.8, 6.9 Hz, 1H), 2.32 (s, 3H), 1.18 (d, *J* = 6.9 Hz, 6H); ^13^C NMR (101 MHz, DMSO-d_6_) δ 174.6, 154.7, 154.2, 153.7, 147.5, 146.4, 140.9, 135.4, 135.2, 133.4, 124.4, 123.0, 121.6, 118.7, 117.4, 32.9, 23.6; HRMS (ESI) m/z [M+H]^+^calcd for C_20_H_21_N_2_O_4_S: 385.1217, found: 385.1198.

**(*E*)-2-chloro-4-fluoro-*N'*-((6-isopropyl-4-oxo-4*H*-chromen-3-yl)methylene)benzenesulfonohydrazide (G22)**

White solid, m.p.210−211°C, yield 61%; ^1^H NMR (500 MHz, DMSO-d_6_) δ 12.12 (s, 1H), 8.40 (s, 1H), 8.14 (d, *J* = 0.6 Hz, 1H), 8.08 (dd, *J* = 9.0, 5.9 Hz, 1H), 7.85 – 7.83 (m, 1H), 7.69 (dd, *J* = 7.9, 3.8, 1.5 Hz, 2H), 7.54 (d, *J* = 8.7 Hz, 1H), 7.45 – 7.40 (m, 1H), 3.05 – 2.95 (m, 1H), 1.18 (d, *J* = 6.9 Hz, 6H); ^13^C NMR (101 MHz, DMSO-d_6_) δ 175.2, 163.8, 154.7, 146.9, 140.2, 133.9, 123.5, 122.1, 119.1, 118.0, 33.4, 24.1; HRMS (ESI) m/z [M+H]^+^calcd for C_19_H_17_N_2_O_4_ClFS: 423.0576, found: 423.0555.

**(*E*)-3,4-dichloro-*N'*-((6-isopropyl-4-oxo-4*H*-chromen-3-yl)methylene)benzenesulfonohydrazide (G23)**

White solid, m.p.203−204°C, yield 65%; ^1^H NMR (500 MHz, DMSO-d_6_) δ 9.92 (s, 1H), 7.43 (s, 1H), 6.94 (d, *J* = 2.5 Hz, 2H), 6.82 (d, *J* = 6.7 Hz, 1H), 6.81 – 6.77 (m, 2H), 6.69 (d, *J* = 6.9 Hz, 1H), 6.59 (d, *J* = 6.9 Hz, 1H), 2.93 (hept, *J* = 5.5 Hz, 1H), 1.48 (d, *J* = 5.5 Hz, 6H); ^13^C NMR (101 MHz, DMSO-d_6_) δ 147.8, 131.8, 131.4, 125.2, 120.9, 119.2, 117.1, 114.8, 113.8, 113.5, 111.1, 110.0, 106.4, 105.3, 103.0, 101.9, 34.4, 27.0; HRMS (ESI) m/z [M+H]^+^calcd for C_19_H_17_N_2_O_4_Cl_2_S: 439.0281, found: 439.0259.

**(*E*)-3,4-difluoro-*N'*-((6-isopropyl-4-oxo-4*H*-chromen-3-yl)methylene)benzenesulfonohydrazide (G24)**

White solid, m.p.206−207°C, yield 69%; ^1^H NMR (500 MHz, DMSO-d_6_) δ 11.69 (s, 1H), 8.64 (s, *J* = 0.4 Hz, 1H), 8.00 (s, *J* = 0.6 Hz, 1H), 7.92 (t, *J* = 8.6 Hz, 1H), 7.84 (d, *J* = 2.3 Hz, 1H), 7.75 (d, *J* = 8.2 Hz, 1H), 7.70 (d, *J* = 10.9 Hz, 1H), 7.67 (d, *J* = 10.3 Hz, 1H), 7.58 (d, *J* = 8.7 Hz, 1H), 3.06 – 2.96 (m, 1H), 1.19 (d, *J* = 6.9 Hz, 6H); ^13^C NMR (101 MHz, DMSO-d_6_) δ 175.2, 155.2, 154.7, 146.9, 141.2, 133.9, 123.5, 122.1, 119.2, 117.9, 33.4, 24.1; HRMS (ESI) m/z [M+H]^+^calcd for C_19_H_17_N_2_O_4_F_2_S: 407.0872, found: 407.0852.

**(*E*)-*N'*-((6-isopropyl-4-oxo-4*H*-chromen-3-yl)methylene)benzenesulfonohydrazide (G25)**

White solid, m.p.244−245°C, yield 53%; ^1^H NMR (500 MHz, DMSO-d_6_) δ 11.59 (s, 1H), 8.58 (d, *J* = 0.5 Hz, 1H), 7.99 (d, *J* = 0.6 Hz, 1H), 7.88 – 7.82 (m, 3H), 7.70 (d, *J* = 8.8 Hz, 1H), 7.63 (t, *J* = 7.3 Hz, 1H), 7.58 (dd, *J* = 8.1, 6.9 Hz, 3H), 3.00 (dt, *J* = 13.7, 6.9 Hz, 1H), 1.18 (d, *J* = 6.9 Hz, 6H); ^13^C NMR (101 MHz, DMSO-d_6_) δ 147.7, 131.3, 131.2, 125.0, 119.8, 119.0, 114.6, 114.4, 111.3, 109.6, 106.3, 105.1, 102.8, 101.9, 34.2, 26.8; HRMS (ESI) m/z [M+H]^+^calcd for C_19_H_19_N_2_O_4_S: 371.1060, found: 371.1042.

**(*E*)-4-chloro-*N'*-((6-isopropyl-4-oxo-4*H*-chromen-3-yl)methylene)benzenesulfonohydrazide (G26)**

White solid, m.p.171−172°C, yield 65%; ^1^H NMR (500 MHz, DMSO-d_6_) δ 11.65 (s, 1H), 8.60 (d, *J* = 0.6 Hz, 1H), 7.99 (d, *J* = 0.6 Hz, 1H), 7.87 (d, *J* = 2.0 Hz, 1H), 7.85 (d, *J* = 2.1 Hz, 1H), 7.84 (d, *J* = 2.3 Hz, 1H), 7.70 (d, *J* = 8.7 Hz, 1H), 7.66 (d, *J* = 2.1 Hz, 1H), 7.64 (d, *J* = 2.0 Hz, 1H), 7.58 (d, *J* = 8.7 Hz, 1H), 3.05 – 2.96 (m, 1H), 1.19 (s, 3H), 1.18 (s, 3H); ^13^C NMR (101 MHz, DMSO-d_6_) δ 174.7, 154.45, 154.2, 146.4, 140.4, 138.1, 137.6, 133.4, 129.5, 129.2, 123.0, 121.6, 118.6, 117.5, 32.9, 23.6 ; HRMS (ESI) m/z [M+H]^+^calcd for C_19_H_18_N_2_O_4_ClS: 405.0670, found: 405.0649.


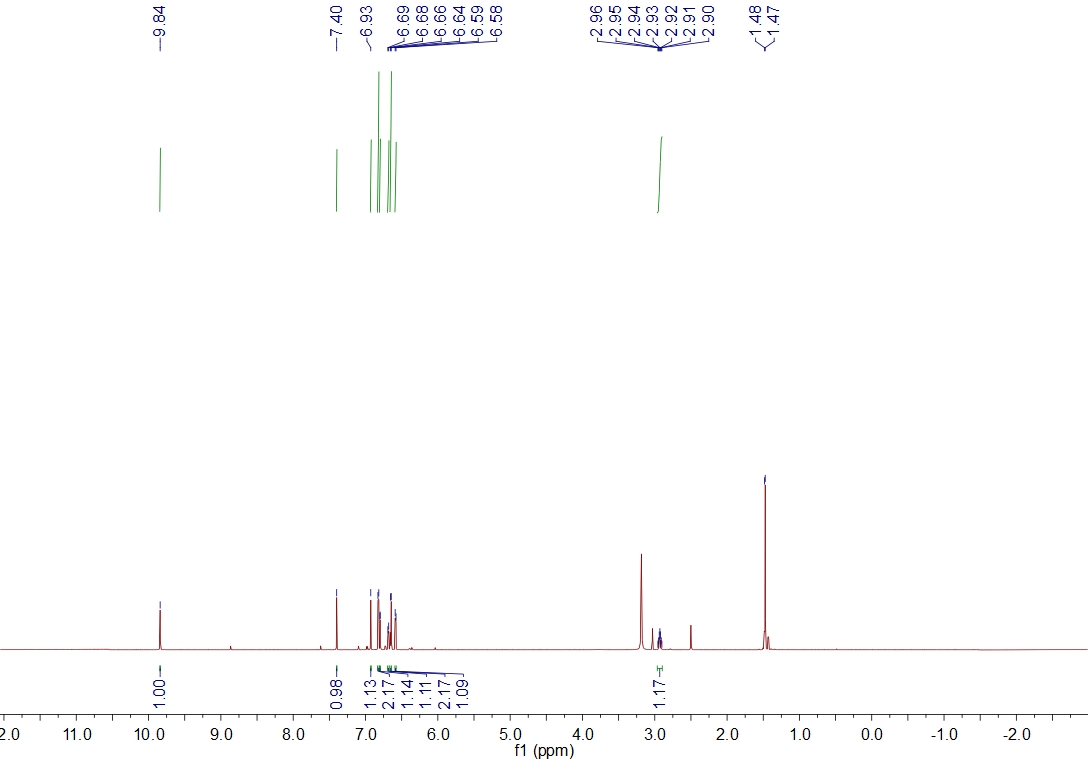


**Fig. S1** ^1^H NMR sprectrum of title compound **G15**


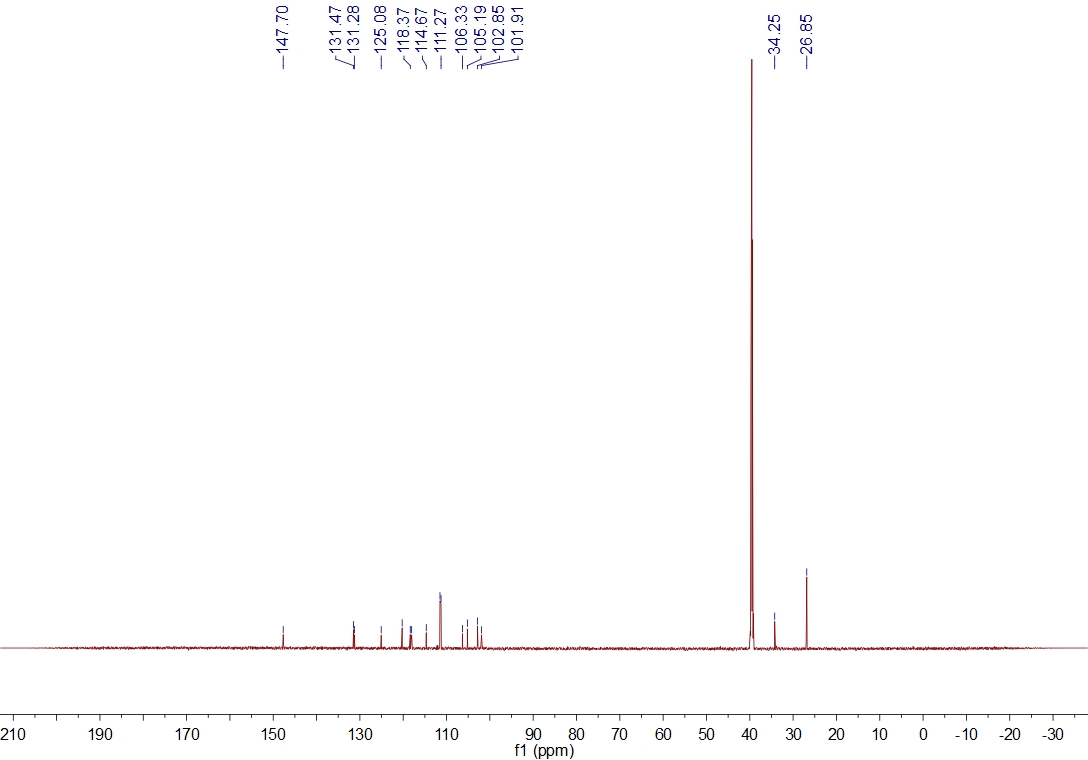


**Fig. S2**^13^C NMR sprectrum of title compound **G15**

**Fig. S3** HRMS sprectrum of title compound **G15**


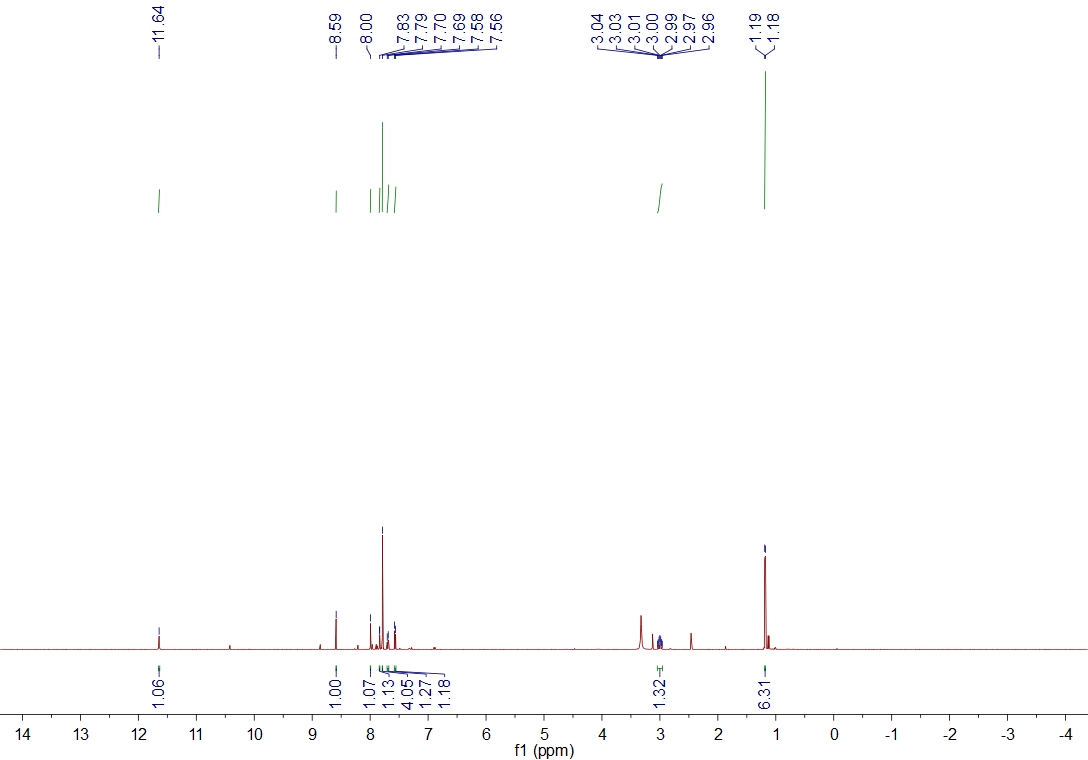


**Fig. S4** ^1^H NMR sprectrum of title compound **G16**


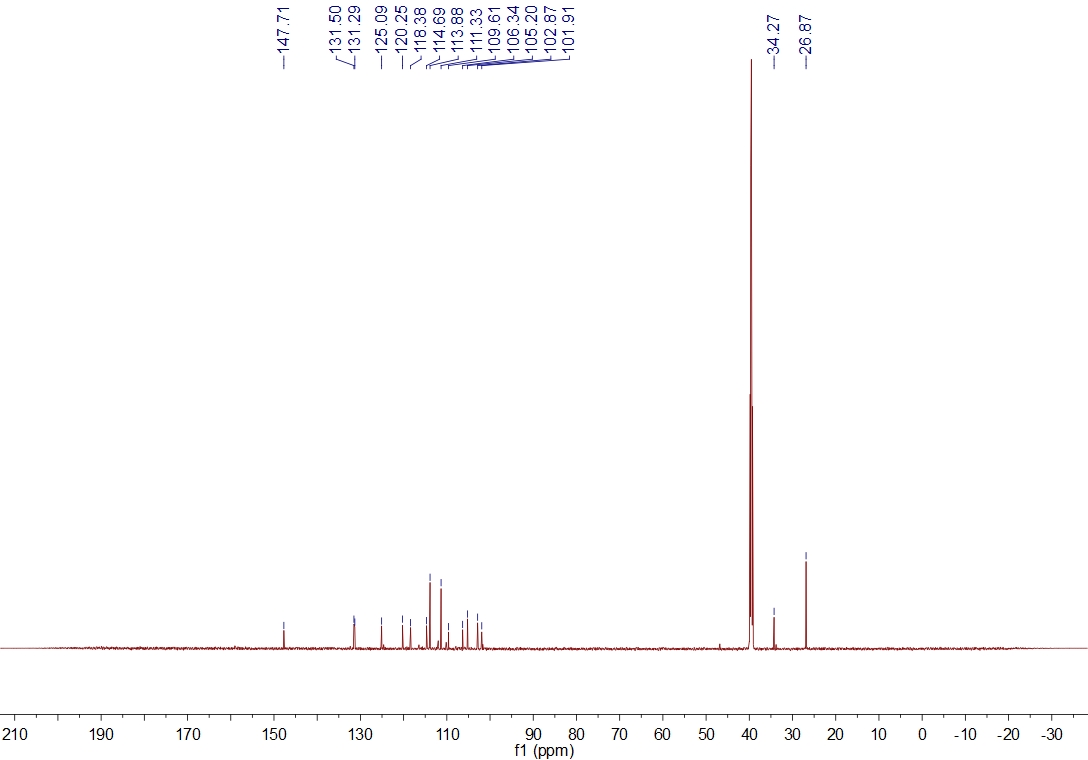


**Fig. S5**^13^C NMR sprectrum of title compound **G16**

**Fig. S6** HRMS sprectrum of title compound **G16**


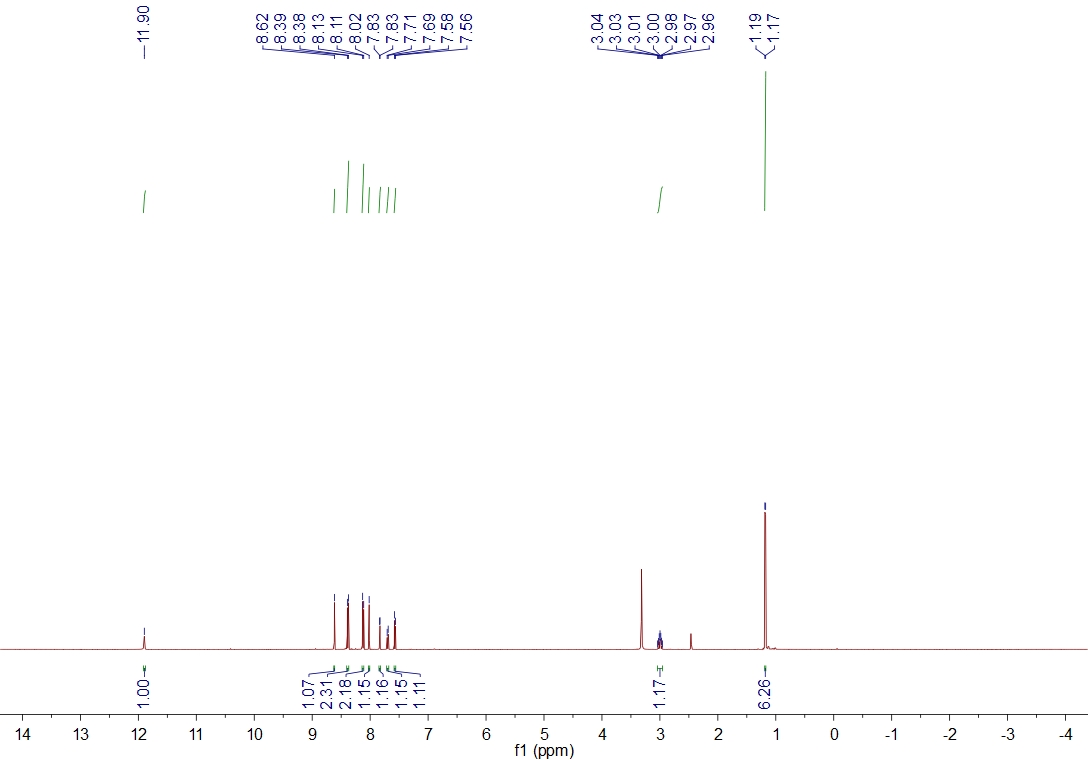


**Fig. S7** ^1^H NMR sprectrum of title compound **G17**


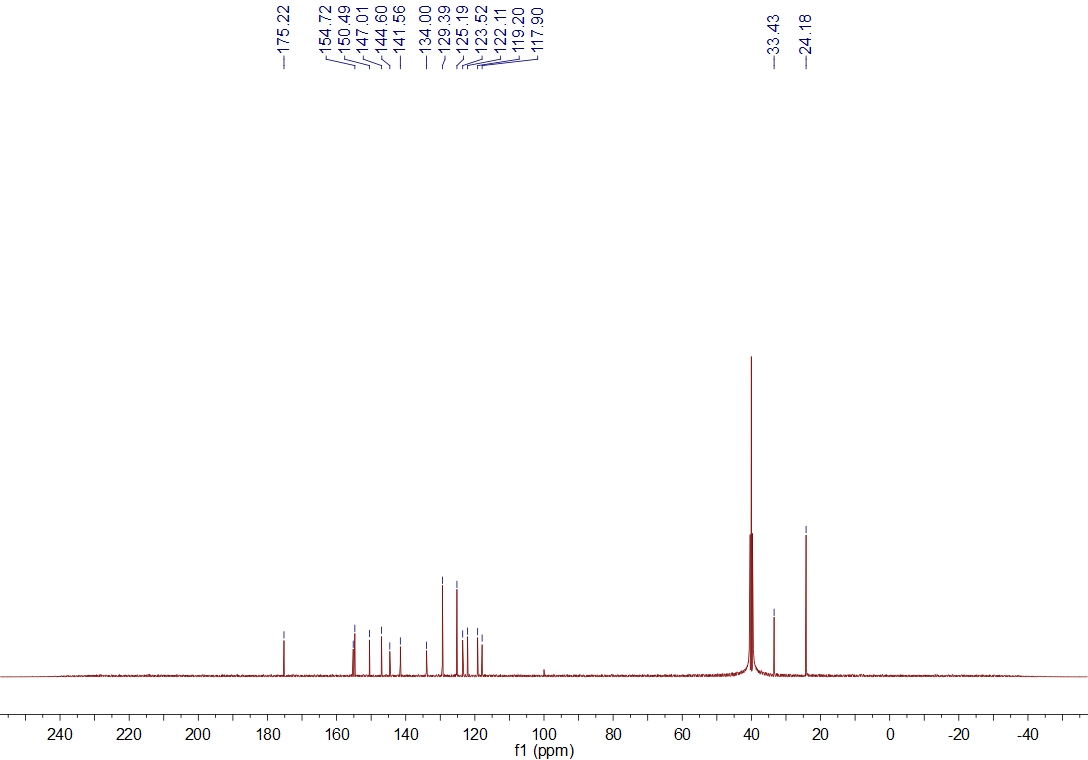


**Fig. S8**^13^C NMR sprectrum of title compound **G17**

**Fig. S9** HRMS sprectrum of title compound **G17**


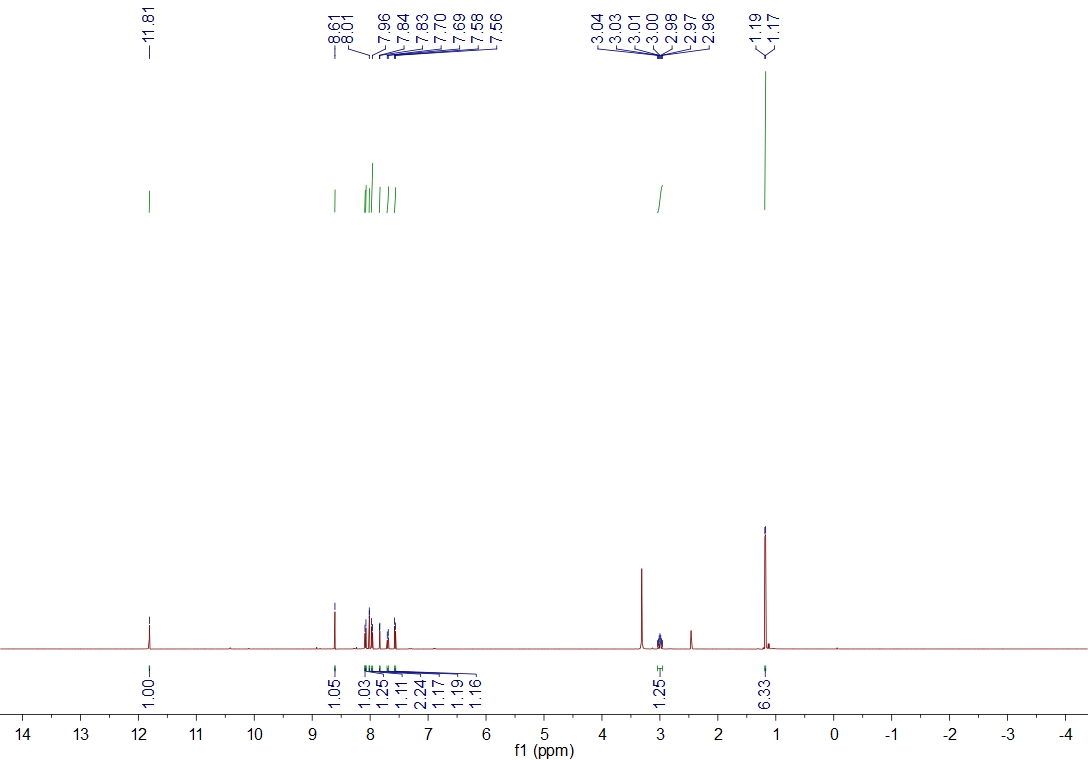


**Fig. S10** ^1^H NMR sprectrum of title compound **G18**


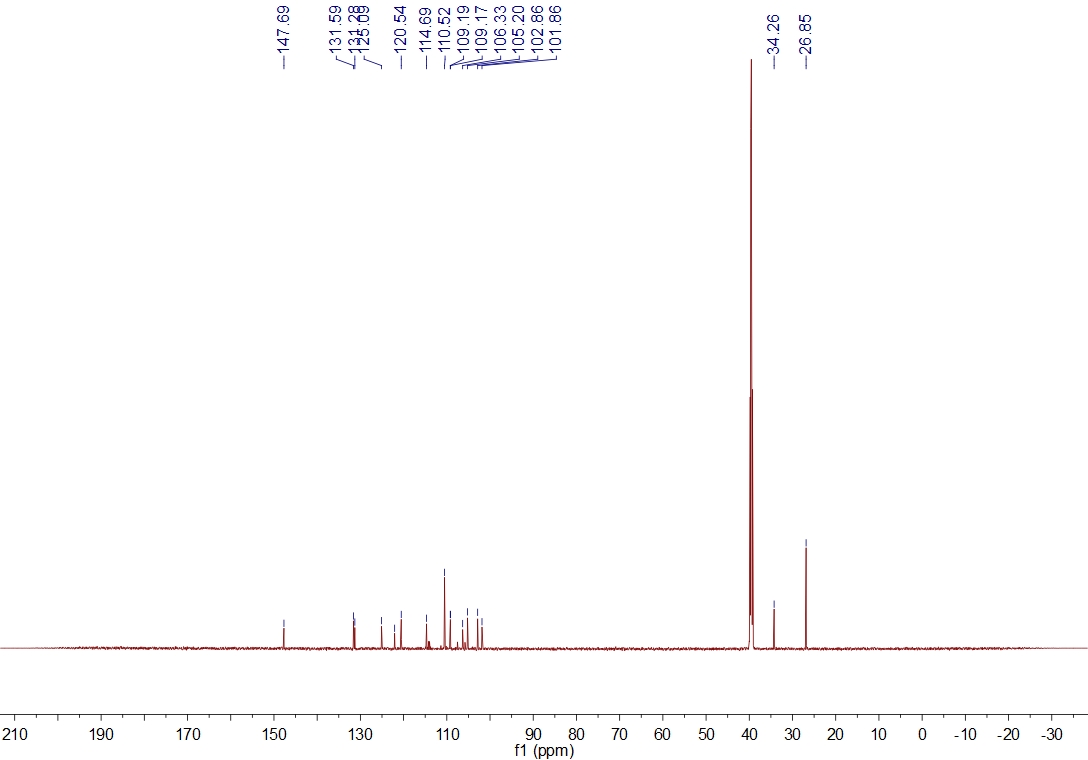


**Fig. S11**^13^C NMR sprectrum of title compound **G18**

**Fig. S12** HRMS sprectrum of title compound **G18**


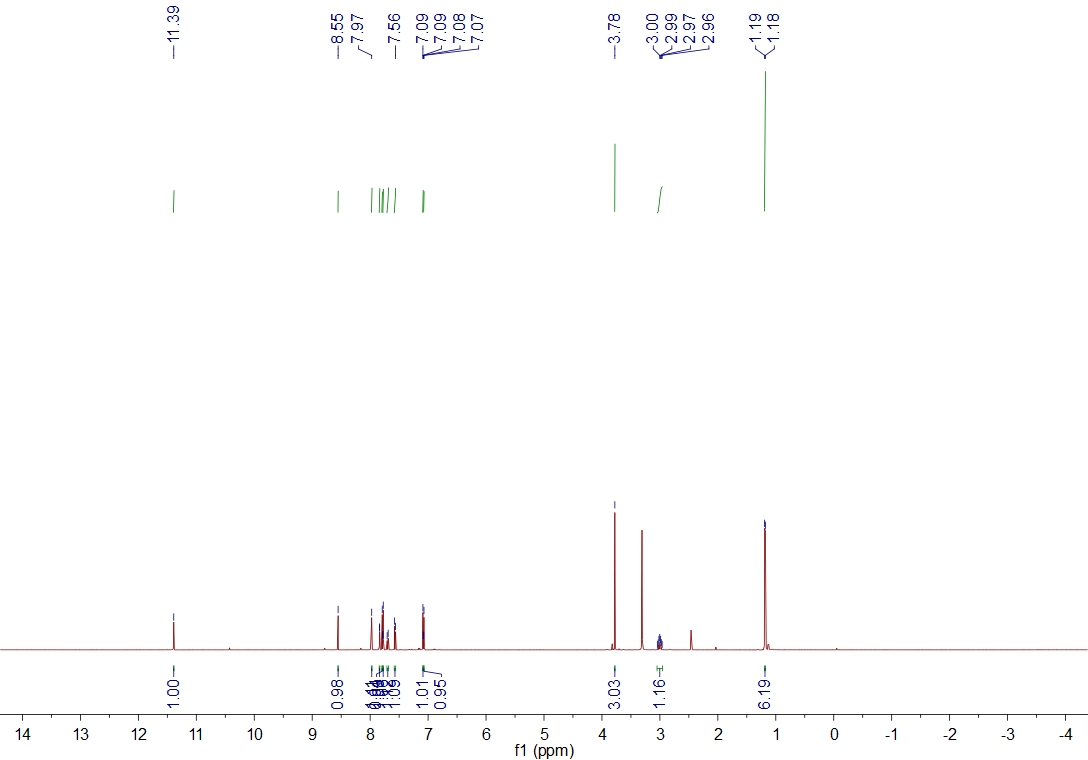


**Fig. S13** ^1^H NMR sprectrum of title compound **G19**


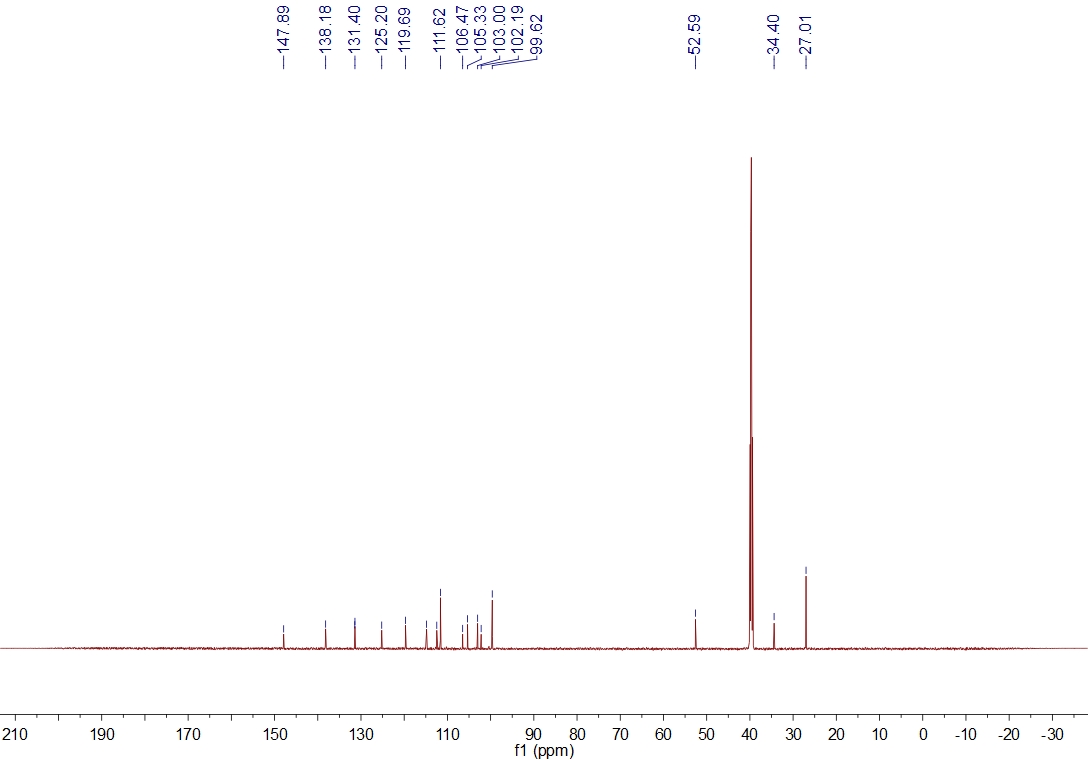


**Fig. S14**^13^C NMR sprectrum of title compound **G19**

**Fig. S15** HRMS sprectrum of title compound **G19**


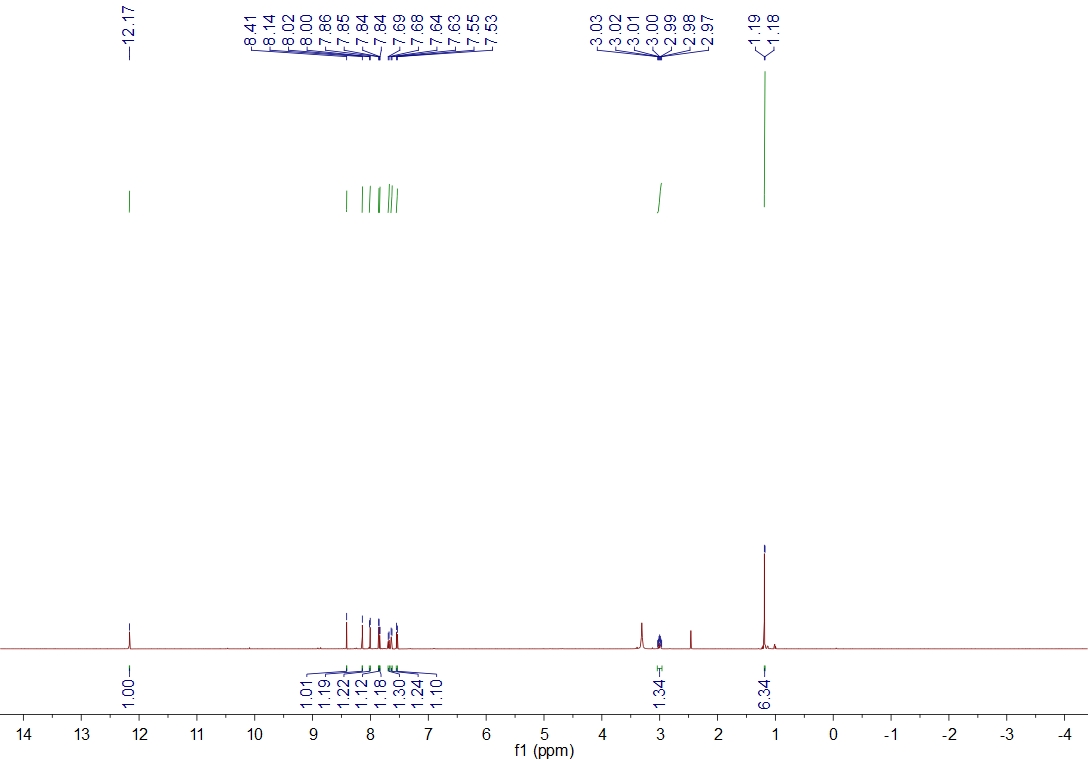


**Fig. S16** ^1^H NMR sprectrum of title compound **G20**


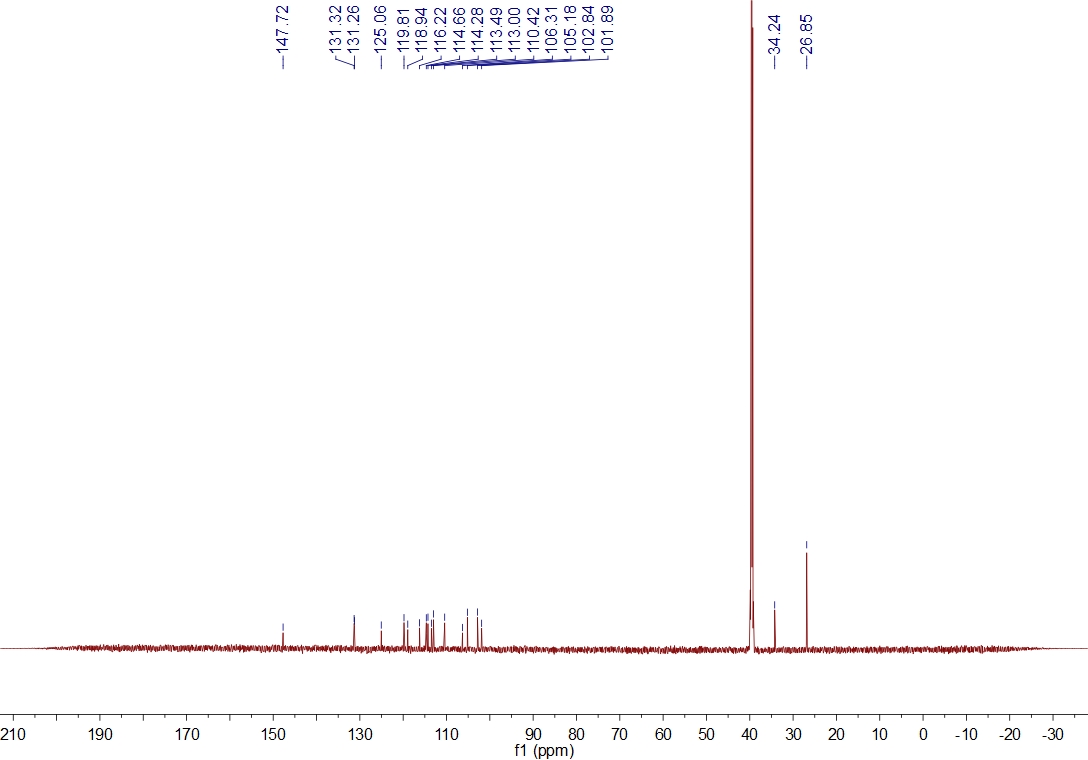


**Fig. S17**^13^C NMR sprectrum of title compound **G20**

**Fig. S18** HRMS sprectrum of title compound **G20**


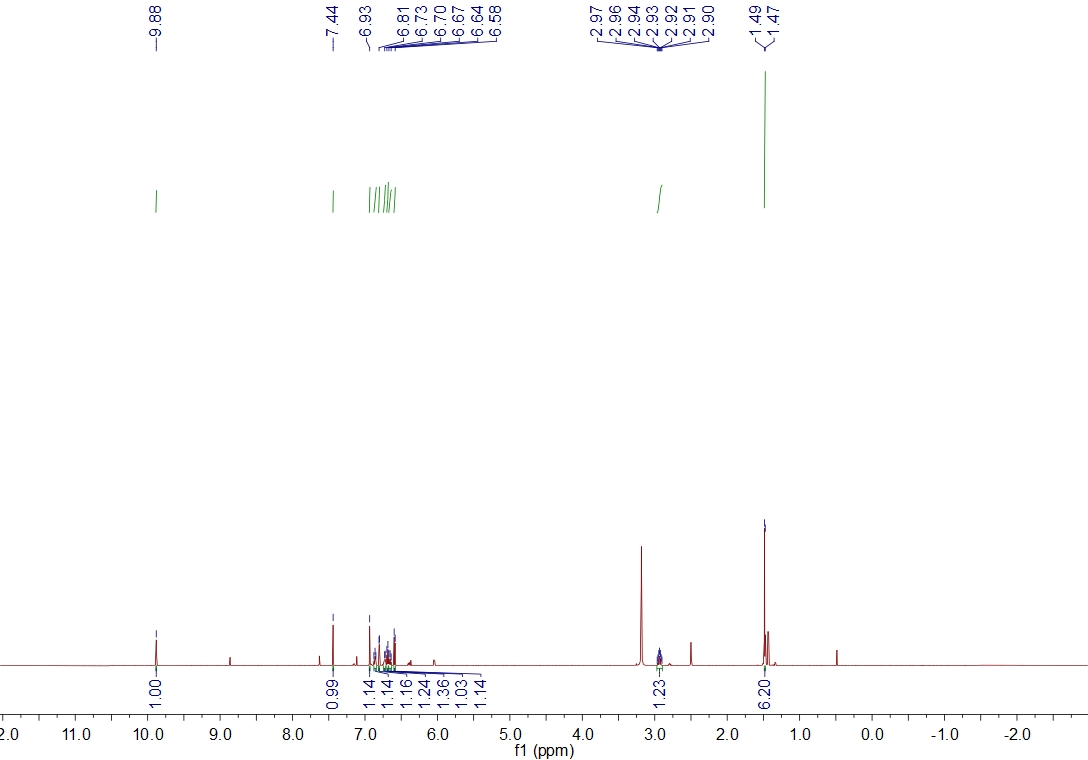


**Fig. S19** ^1^H NMR sprectrum of title compound **G21**


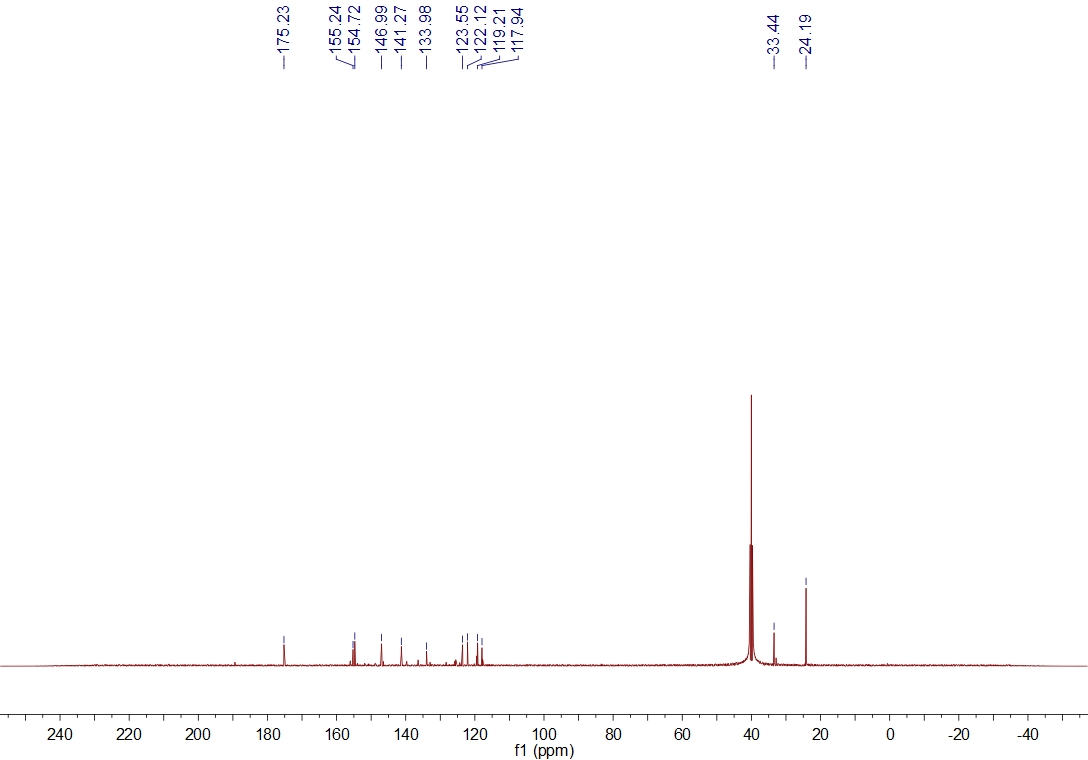


**Fig. S20**^13^C NMR sprectrum of title compound **G21**

**Fig. S21** HRMS sprectrum of title compound **G21**


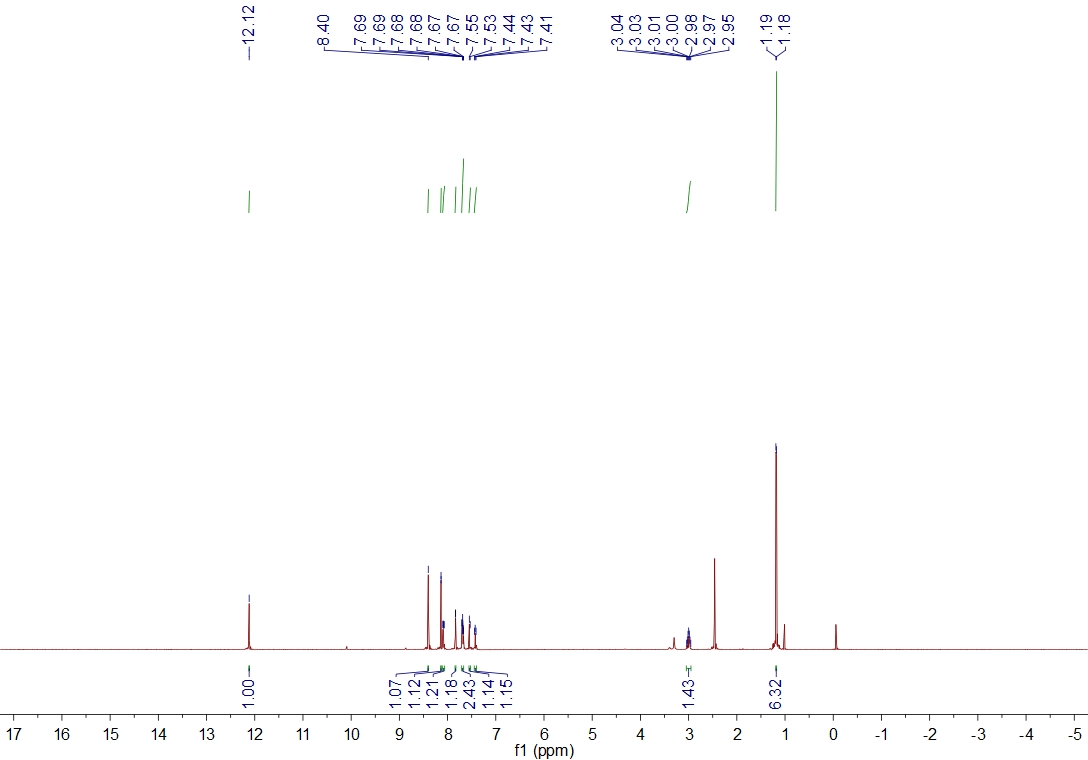


**Fig. S22** ^1^H NMR sprectrum of title compound **G22**


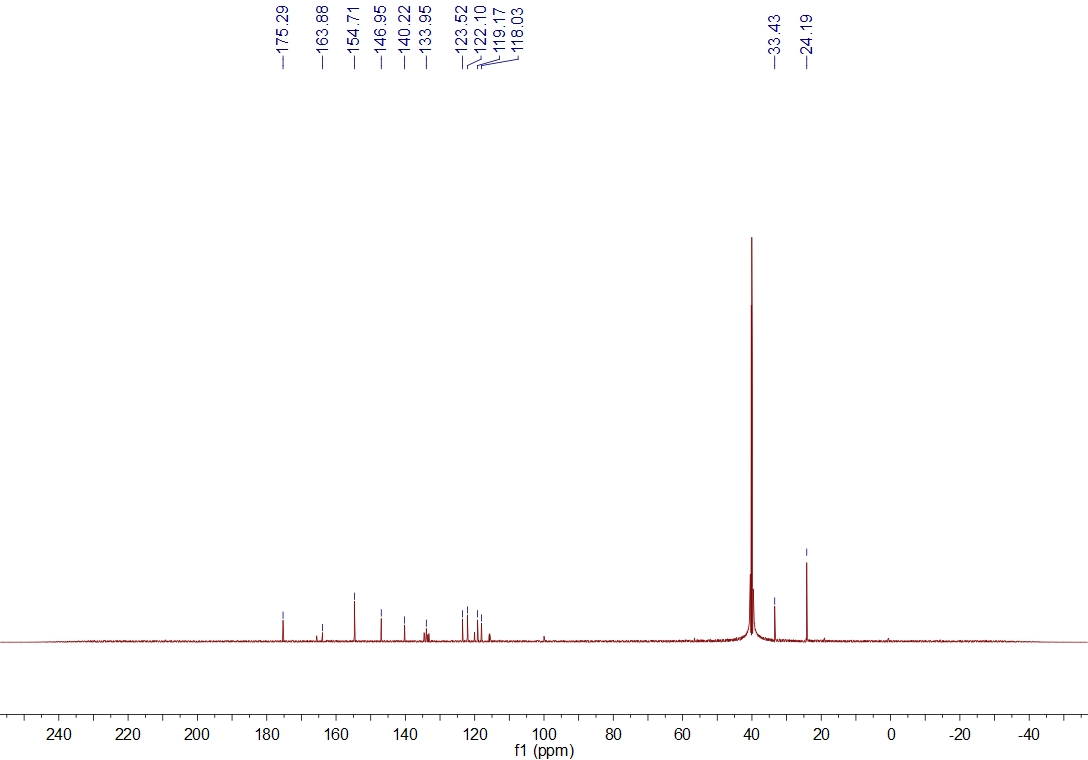


**Fig. S23**^13^C NMR sprectrum of title compound **G22**

**Fig. S24** HRMS sprectrum of title compound **G22**


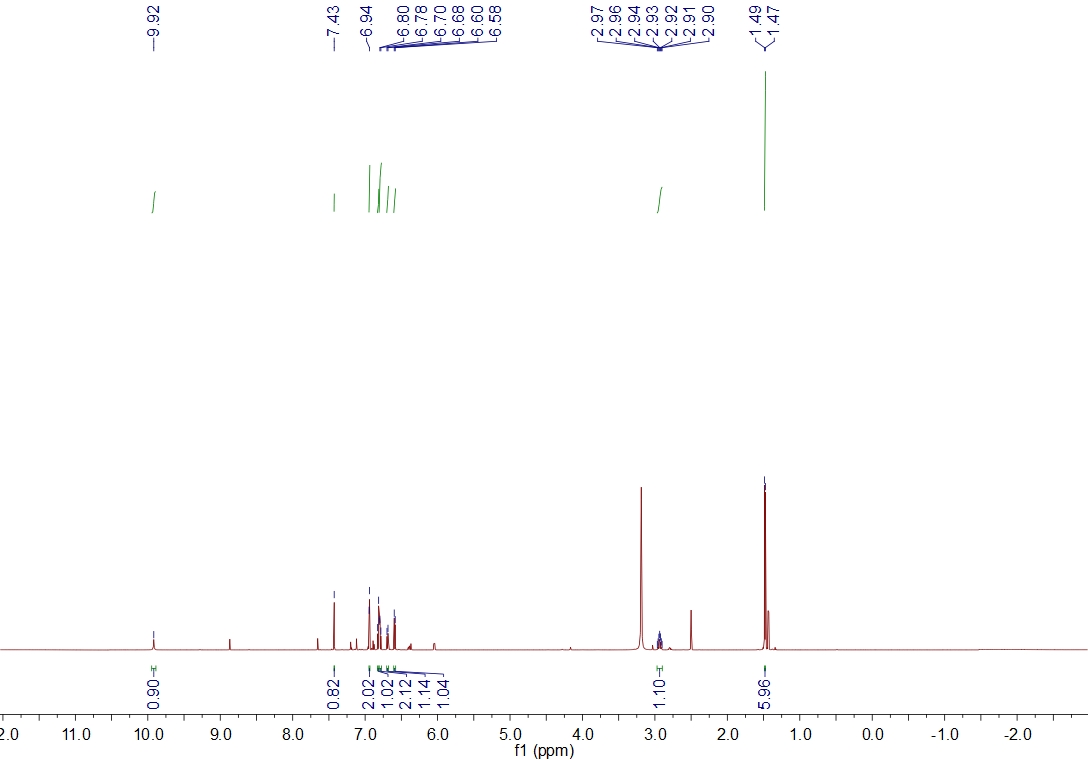


**Fig. S25** ^1^H NMR sprectrum of title compound **G23**


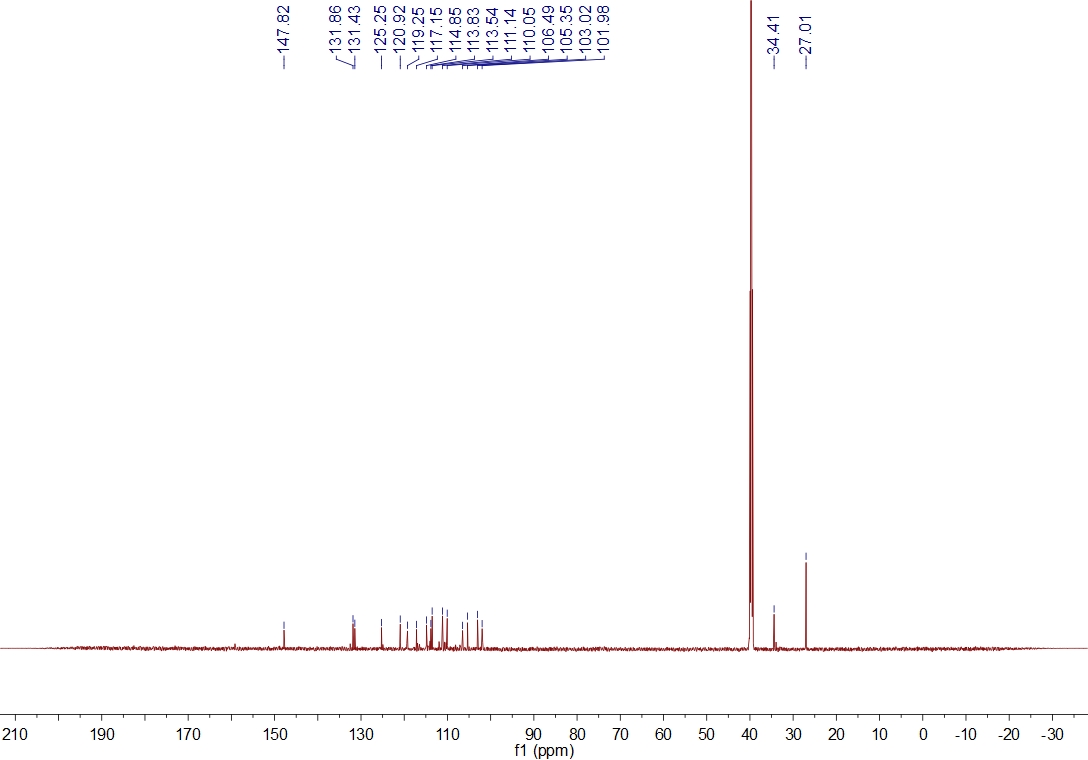


**Fig. S26**^13^C NMR sprectrum of title compound **G23**

**Fig. S27** HRMS sprectrum of title compound **G23**


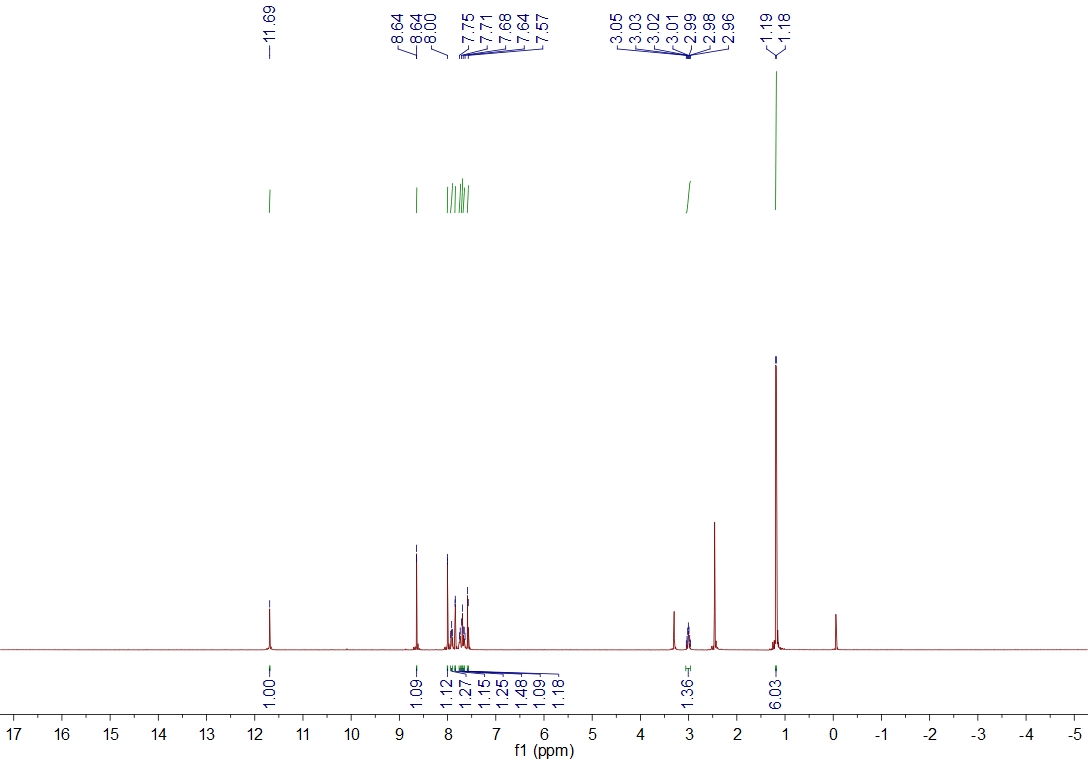


**Fig. S28** ^1^H NMR sprectrum of title compound **G24**


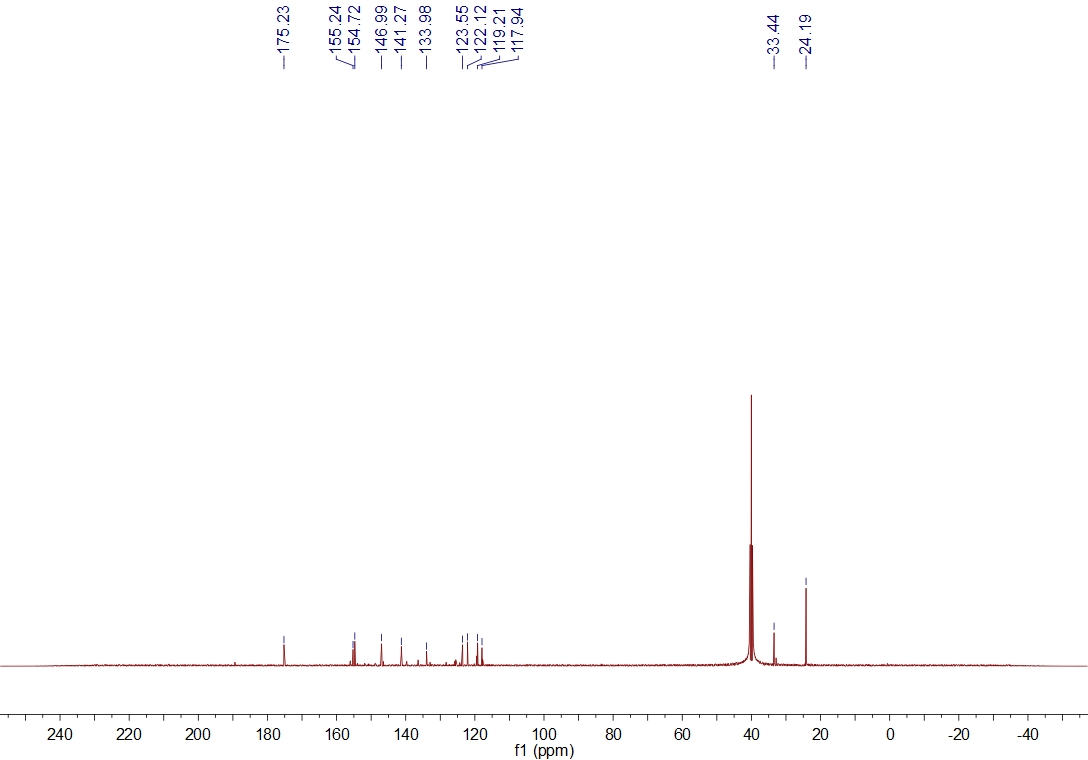


**Fig. S29**^13^C NMR sprectrum of title compound **G24**

**Fig. S30** HRMS sprectrum of title compound **G24**


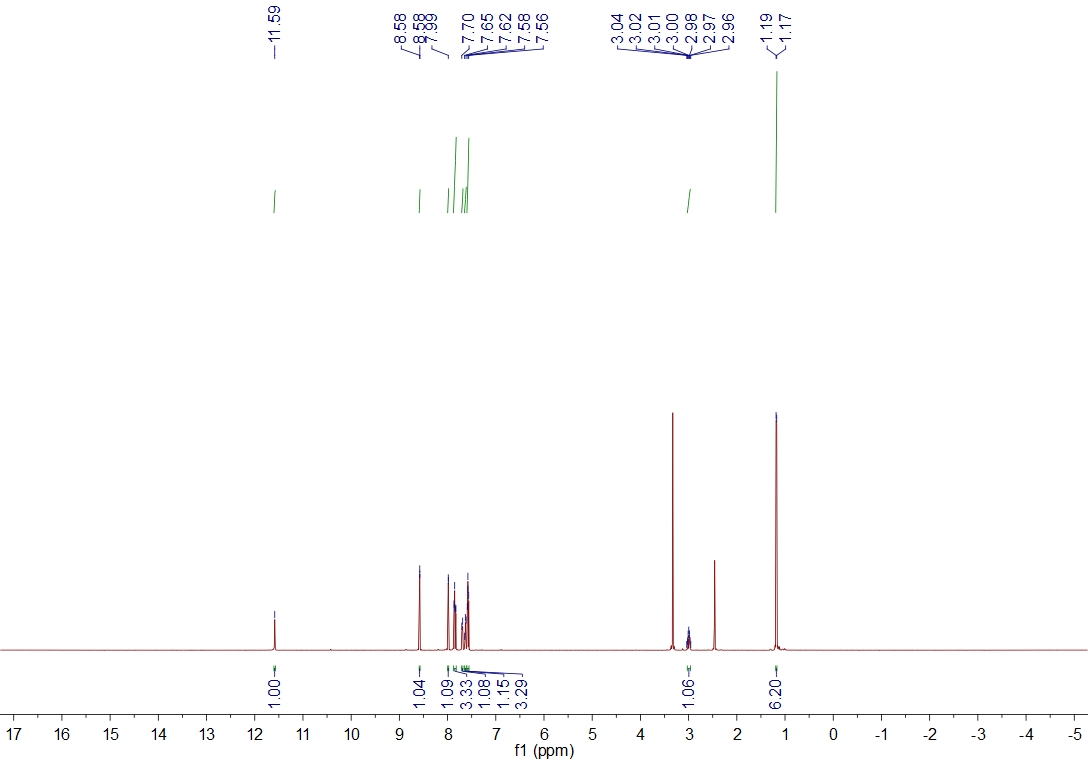


**Fig. S31** ^1^H NMR sprectrum of title compound **G25**


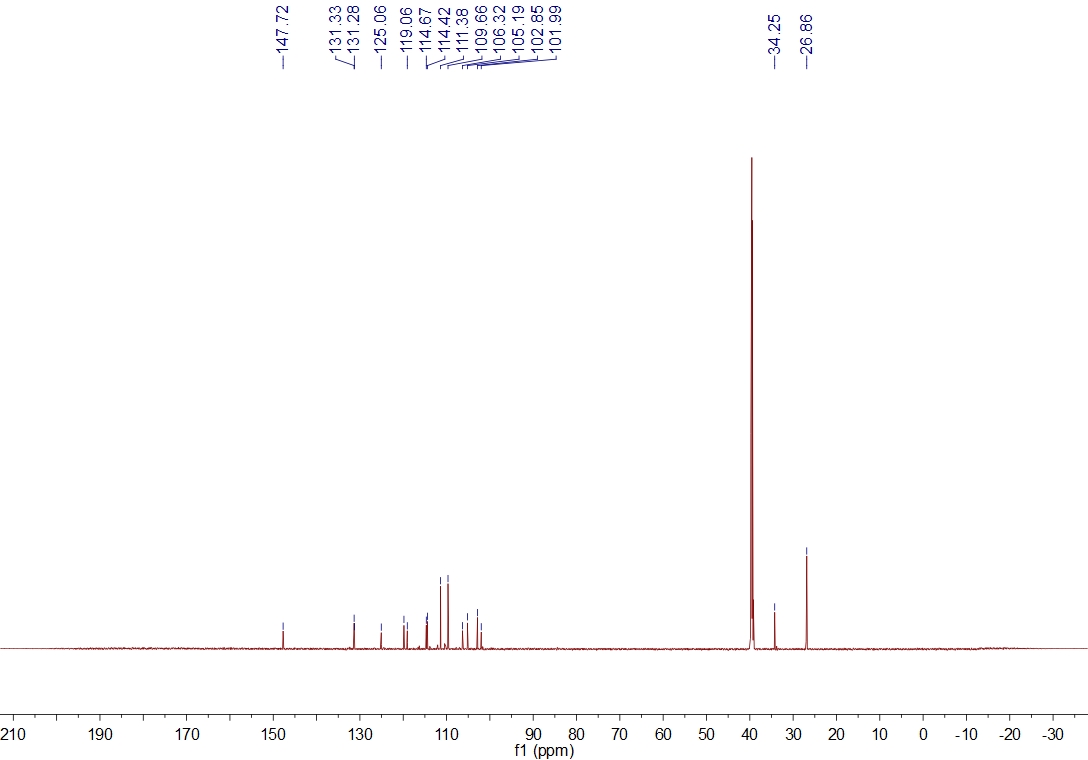


**Fig. S32**^13^C NMR sprectrum of title compound **G25**

**Fig. S33** HRMS sprectrum of title compound **G25**


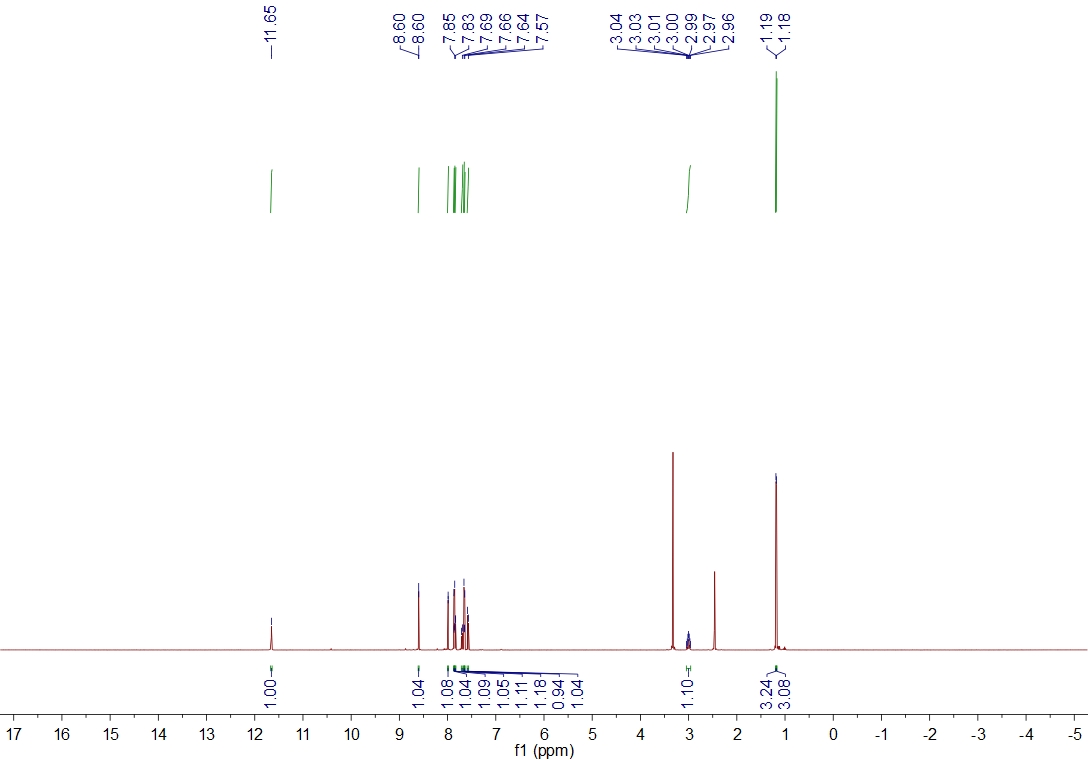


**Fig. S34** ^1^H NMR sprectrum of title compound **G26**


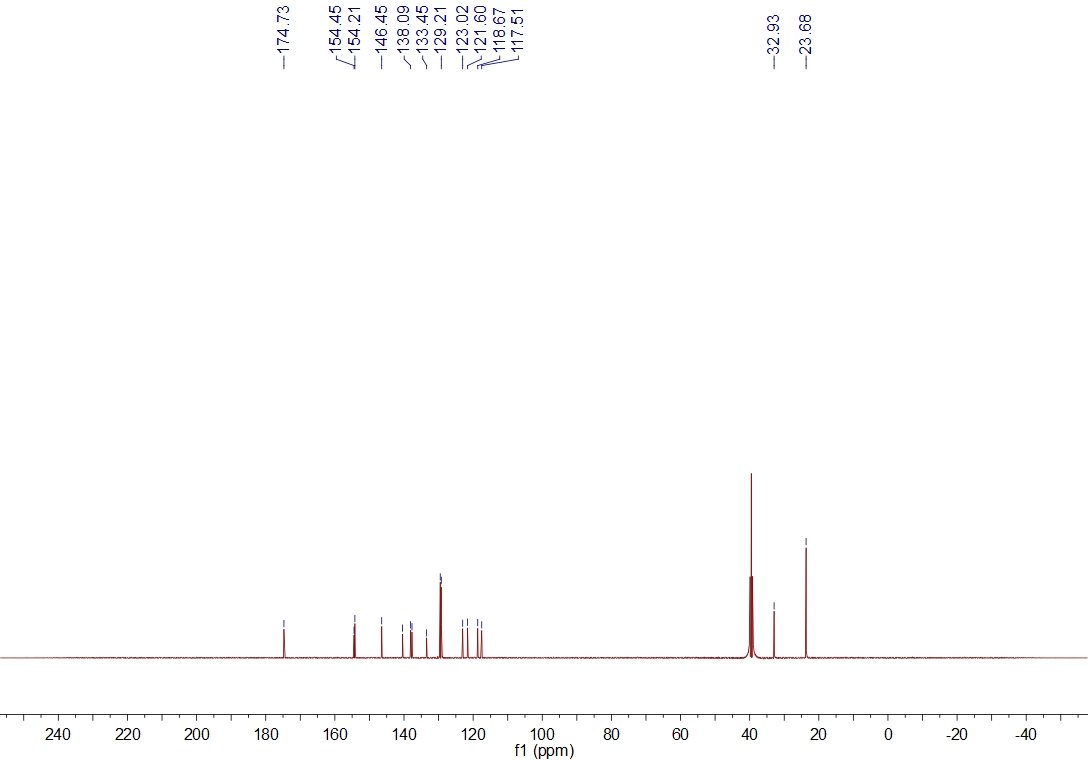


**Fig. S35**^13^C NMR sprectrum of title compound **G26**

**Fig. S36** HRMS sprectrum of title compound **G26**
